# Profiling the Interaction Mechanism of Quinoline/Quinazoline Derivatives as MCHR1 Antagonists: An *in Silico* Method

**DOI:** 10.3390/ijms150915475

**Published:** 2014-09-01

**Authors:** Mingwei Wu, Yan Li, Xinmei Fu, Jinghui Wang, Shuwei Zhang, Ling Yang

**Affiliations:** 1 Key Laboratory of Industrial Ecology and Environmental Engineering (MOE), Dalian University of Technology, Dalian 116024, China; E-Mails: audi.lg@163.com (M.W.); jhwang_dlut@163.com (J.W.); zswei@dlut.edu.cn (S.Z.); 2 State Key Laboratory of Fine Chemicals, Dalian University of Technology, Dalian 116024, China; E-Mail: fuxinmei@dlut.edu.cn; 3 Laboratory of Pharmaceutical Resource Discovery, Dalian Institute of Chemical Physics, Graduate School of the Chinese Academy of Sciences, Dalian 116023, China; E-Mail: yling@dicp.ac.cn

**Keywords:** MCHR1, 3D-QSAR, molecular docking, MD simulation

## Abstract

Melanin concentrating hormone receptor 1 (MCHR1), a crucial regulator of energy homeostasis involved in the control of feeding and energy metabolism, is a promising target for treatment of obesity. In the present work, the up-to-date largest set of 181 quinoline/quinazoline derivatives as MCHR1 antagonists was subjected to both ligand- and receptor-based three-dimensional quantitative structure–activity (3D-QSAR) analysis applying comparative molecular field analysis (CoMFA) and comparative molecular similarity indices analysis (CoMSIA). The optimal predictable CoMSIA model exhibited significant validity with the cross-validated correlation coefficient (*Q*^2^) = 0.509, non-cross-validated correlation coefficient (*R*^2^_ncv_) = 0.841 and the predicted correlation coefficient (*R*^2^_pre__d_) = 0.745. In addition, docking studies and molecular dynamics (MD) simulations were carried out for further elucidation of the binding modes of MCHR1 antagonists. MD simulations in both water and lipid bilayer systems were performed. We hope that the obtained models and information may help to provide an insight into the interaction mechanism of MCHR1 antagonists and facilitate the design and optimization of novel antagonists as anti-obesity agents.

## 1. Introduction

Obesity, a chronic disease, is correlated with an inappropriate balance between energy intake and expenditure [1]. It has been gradually developed into an alarming pandemic affecting a huge population worldwide, especially in the western countries [2]. No longer regarded as a cosmetic problem, obesity is emerging as a major risk factor for a number of cardiovascular and metabolic disorders such as hypertension, type 2 diabetes, dyslipidemia, atherosclerosis, and certain types of cancers [3]. Furthermore, some obese patients suffer from psychosocial discrimination, which may cause depression and anxiety. The rising prevalence of obesity coupled with its increased complications results in not only high mortality and morbidity rates but also a huge economic burden [4].

Many biological anti-obesity targets have been investigated including centrally modulated satiety and hunger regulating systems [5], among which melanin concentrating hormone (MCH) and its receptors attract extensive attention. MCH, a cyclic 19-amino-acid neuropeptide, was primarily isolated from the pituitary gland of the salmon as a hormone responsible for skin pigmentation [6]. MCH was subsequently found to be present in mammals [7], and its amino acid sequence is highly conserved in fishes, rats, and humans [8]. MCH is expressed mainly in neurons in the lateral hypothalamus and *Zona incerta* that project widely into other regions of the brain [9]. Several early studies have been carried out and published regarding the role MCH plays in the control of feeding and energy metabolism. After injection in the central nervous system (CNS) [10] in mice, MCH stimulates food intake, thus increasing body weight [11] and acting as an important mediator of energy homeostasis [12]. Intracerebroventricular injection of MCH in mice also leads to a dose-dependent increase in food intake [13]. Genetically altered mice over-expressing MCH demonstrate similar traits and are prone to weight gain, insulin resistance and obesity when fed a high fat diet [14]. On the contrary, mice that are lack the MCH gene display hyperactivity and a lean phenotype and are resistant to diet-induced obesity [15]. The biological function of MCH is mediated by G protein-coupled receptors (GPCRs) located in the CNS, and up to now two receptor subtypes, melanin concentrating hormone receptor 1 (MCHR1) and MCHR2, have been identified [16]. MCHRs pertain to the class A family of GPCRs, which are integral membrane proteins containing seven transmembrane helices [17]. MCHR1, ubiquitous to all vertebrates, has received most attention based on its availability for suitable animal models to test its neurobiological functions. Rodents lack MCHR2, and the biological function of MCHR2 remains unclear so far [16], which renders it difficult to determine its functional importance. It is generally accepted that MCHR1 is involved in the neuronal regulation of food consumption. In accordance with this, transgenic mice with an ablation of the gene encoding MCHR1 maintain elevated metabolic rates and keep lean despite hyperphagia on a normal diet [15]. Collectively, these facts indicate that MCHR1 is a crucial regulator of energy homeostasis and suggest the positive role of MCHR1 antagonists as anti-obesity therapeutic agents. In addition, it is notable that MCHR1 antagonists might find an additional usage in the treatment of anxiety and mood disorders for their anxiolytic and antidepressant effects in some animal models [18]. However, possibly due to the existence of more effective therapies as well as less conclusive animal data, development activities related to MCHR1 antagonists within the depression/anxiety indication have always lagged behind obesity [19]. Thus the effect of MCHR1 antagonists on mood disorders is no longer discussed in this article.

**Figure 1:**
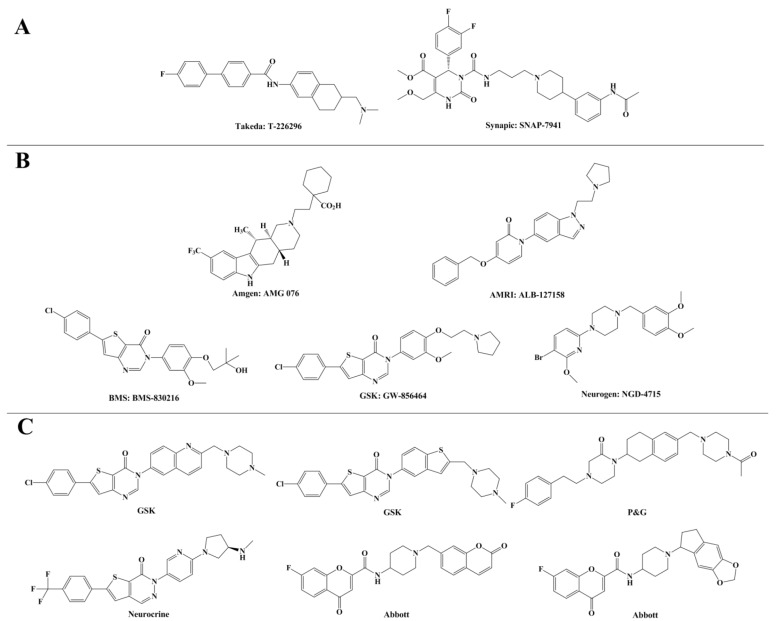
(**A**) Two pioneer melanin concentrating hormone receptor 1 (MCHR1) antagonists; (**B**) Five MCHR1 antagonists in Phase I clinical trials; (**C**) Several potent MCHR1 antagonists with good human ether-a-go-go related gene (hERG) selectivity.

Although the role of MCH and MCHR1 in food intake and energy homeostasis has been of interest for years, it was not until the year 2002 when two seminal papers [20,21] were published supporting the notion of MCHR1 antagonists as potentially useful agents in the treatment of obesity that pharmaceutical and biotechnology corporations joined the competition to develop the first anti-obesity drug. As mentioned, the two pioneer compounds (shown in Figure 1A), T-226296 from Takeda (Osaka, Japan) and SNAP-7941 from Synaptic (Gottingen, Germany), represent the starting point of small molecular MCHR1 antagonists and present the pharmacological evidence of the anti-obesity therapeutic utility of MCHR1 antagonists [22].

In the following decade significant efforts were undertaken to identify and optimize small molecular MCHR1 antagonists. More than 80 medicinal chemistry papers and 100 patent applications have been published due to the intense interest of 23 different companies [22]. Only five candidates depicted in Figure 1B have been tested in human subjects and disclosed to enter Phase I clinical trials so far, none of which has proceeded into the advanced Phase II stage for efficacy and safety studies. The entrance of AMG076 into Phase I trials was reported by the Amgen company (Thousand Oaks, CA, USA), and no progress of its status has been reported since 2005 [23]. Clinical development has also been reported for ALB-127158 developed by AMRI (New York, NY, USA) [19]. This agent also showed tolerability and potential efficacy but it was proclaimed to have stopped with Phase I studies. The most recent antagonist BMS-830216 [24] from BMS (New York, NY, USA) was evaluated in a 28-day Phase I study in obese subjects exhibiting safety and toleration while the antagonist failed to proceed into Phase II studies on account of no observation of reduction in weight or food intake. GlaxoSmithKline thienopyrimidinone compound GW-856464 was found to be a potent MCHR1 antagonist with high selectivity, nevertheless, its low bioavailability precluded further development [25]. The Neurogen MCHR1 antagonist NGD-4715, a piperazine compound, was discontinued for further clinical development though announced to be safe and well tolerated [25]. The contrast between the substantial drug-discovery programs and the limited number of agents progressed into the clinical stage is notable. Besides the traditional challenges in drug design such as absorption, distribution, metabolism and elimination (ADME) and safety profiles, further development of significant numbers of MCH-R1 antagonists has been compromised by potential cardiac liabilities induced by human ether-a-go-go related gene (hERG) channel binding. The high-affinity hERG binding as well as subsequent induced QT interval prolongation possibly result in increased risk of cardiovascular disease, which has led to many approved drugs being withdrawn from the market [23]. Numerous MCHR1 antagonists and conventional hERG agents have one structural element in common: a central scaffold attached to an aryl or heteroaryl moiety and a basic amino group [23]. It is no wonder that a good many effective MCHR1 antagonists are also potent hERG blockers. Hence, further considerable efforts are needed to develop MCHR1 antagonists that are capable of overcoming hERG liabilities while remaining orally active, potent and selective with sufficient brain penetration. Some disclosed preclinical potent antagonists that exhibit good hERG selectivity are listed in Figure 1C.

As an effective and economical method, three-dimensional quantitative structure–activity relationship (3D-QSAR) has been extensively applied in exploration of interaction mechanisms, characterization of action features and prediction of drug activities to help design novel pharmaceuticals [26,27,28]. In the present work, a series of quinoline and quinazoline derivative antagonists attracted our attention, which may improve hERG liability and solubility, and the up-to-date largest dataset based on 181 molecules [29,30,31,32] was used to build 3D-QSAR models. The target-antagonist binding activities were investigated by a combination of 3D-QSAR, docking and molecular dynamics. Due to the consideration that MCHR1 contains seven transmembrane domains, molecular dynamics (MD) simulations were performed not only traditionally with the receptor in water but also with the receptor in a lipid bilayer. We expect that the comprehensive models and inferences obtained may offer helpful references in the development of novel effective MCHR1 antagonists.

## 2. Results and Discussion

### 2.1. Three-Dimensional Quantitative Structure–Activity Relationship (3D-QSAR) Statistical Results

In our present work, ligand-based strategy was carried out in both comparative molecular field analysis (CoMFA) and comparative molecular similarity index analysis (CoMSIA) analyses with the test and training set containing 60 and 121 antagonist molecules, respectively. All combinations of the field descriptors were attempted in order to choose the optimal model. We developed our 3D-QSAR models and assessed the predictive ability by applying partial least squares (PLS) analysis as well as the leave-one-out (LOO) cross-validation method. Several important parameters were obtained, including the cross-validated correlation coefficient (*Q*^2^), non-cross-validated correlation coefficient (*R*^2^_ncv_), the predicted correlation coefficient (*R*^2^_pred_), standard error of estimate (SEE), the optimum number of components (OPN), which was determined by the number of components that yielded the smallest SEE and *F* values. Under normal conditions, values larger F mean that fewer explanatory variables and more target properties are acquired from a model, which implies that the model is more statistically significant [33]. In general, the CoMFA and CoMSIA models with the optimal statistics are determined by the highest *Q*^2^, the lowest SEE and the fewest OPN, which is applied to generate the final model [34]. The statistical results derived from the ligand-based strategy are listed in Table 1.

**Table 1:** Summary of comparative molecular field analysis (CoMFA) and comparative molecular similarity index analysis (CoMSIA) results.

PLS Statistics	CoMFA	CoMSIA
*Q*^2^	0.372	0.509
*R*^2^_ncv_	0.896	0.841
SEE	0.235	0.288
*F*	138.780	100.745
*R*^2^_pred_	0.544	0.745
SEP	0.576	0.507
OPN	7	6
Contribution (%)		
Steric	54.8	14.8
Electrostatic	45.2	–
Hydrophobic	–	39.0
H-bond donor	–	23.1
H-bond acceptor	–	23.1

For CoMFA analysis, the model employing both the steric and electrostatic field descriptors presents a so-so statistical result providing *Q*^2^ = 0.372 with OPN of 7, *R*^2^_ncv_ = 0.896, SEP = 0.576, SEE = 0.235, *F* = 138.780 and the contribution of steric feature (54.8%) is slightly higher than that of the electrostatic feature (45.2%). In general, a model with cross-validated *Q*^2^ > 0.5 is indicative of a good predictive model, which manifests that the CoMFA model obtained tends to be statistically unacceptable in terms of prediction capacity in spite of its relatively high value of *R*^2^_ncv_.

**Figure 2:**
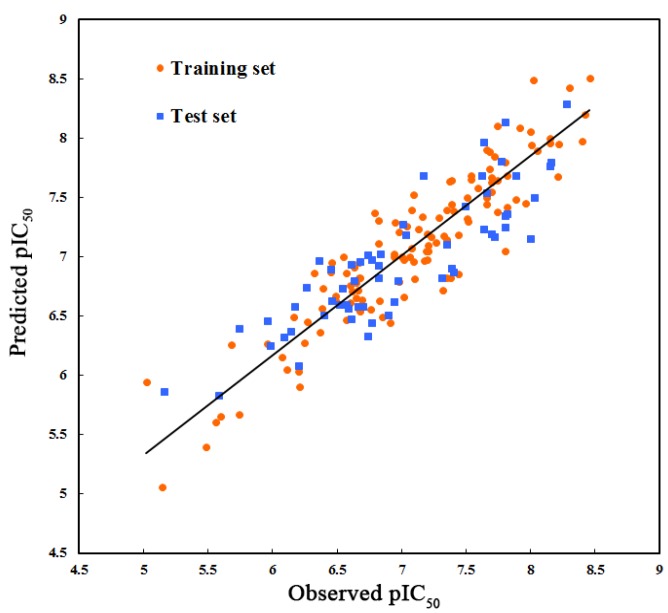
The ligand-based correlation plot of the predicted versus the actual pIC_50_ values based on the comparative molecular similarity index analysis (CoMSIA) model. The solid line is the regression line for the fitted and predicted bioactivities of training compounds in the optimal CoMSIA model.

In CoMSIA analysis, as the consideration that the field descriptors (steric, electrostatic, hydrophobic, H-bond donor and H-bond acceptor) may be somewhat dependent on each other and the predictive accuracy of the model may be adversely affected [35,36], all 31 combinations of the five parameters were calculated and the optimal one was selected referring to their respective *Q*^2^ and OPN value. Finally, four field descriptors consisting of steric, hydrophobic, H-bond donor fields and H-bond acceptor fields were used to construct the best CoMSIA model, ending up with an acceptable *Q*^2^ value of 0.509 with OPN of six, a high value of 0.841 for *R*^2^_ncv_ as well as a high *F* value of 138.780, which shows its good internal predictive capacity. The predictive correlation coefficient *R*^2^_pred_ of 0.745 demonstrates an appropriate predictive power of the model constructed. As to the relative contribution, the hydrophobic field, which shows the greatest contribution of 0.390 seems to play a crucial role in the binding of antagonists to MCHR1 while the steric contribution is only 14.8% of the variance, accounting for the smallest coefficent. The other two field descriptors, H-bond donor and acceptor, give the same contributions both explaining 23.1%. The correlation plot of the observed pIC_50_ *versus* the predicted data for CoMSIA model is illustrated in Figure 2 with the training set symbolized by orange circles and the test set by blue squares. The predicted pIC_50_ is in accordance with the experimental results, which indicates no systematic errors in the method.

### 2.2. Comparative Molecular Similarity Index Analysis (CoMSIA) Contour Maps Analysis

**Figure 3:**
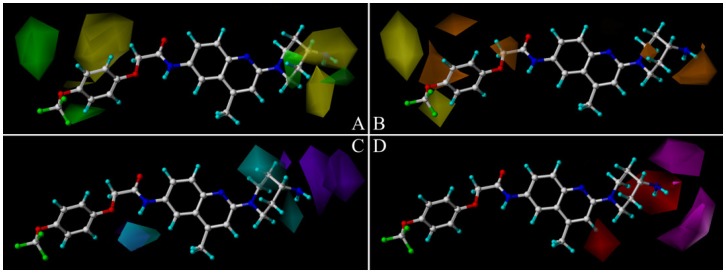
CoMSIA coeff*stddev contour maps in combination with Antagonist **169**. (**A**) Steric fields: green contours represent regions where bulky groups increase the activity, while yellow contours represent regions where bulky groups decrease the activity; (**B**) hydrophobic fields: yellow contours indicate regions where hydrophobic feature favors the activity, while orange contours indicate where hydrophobic feature disfavors the activity; (**C**) H-bond donor fields: cyan contours indicate where H-bond donors are beneficial for the activity, purple contours indicate where H-bond donors are detrimental for the activity; (**D**) H-bond acceptor fields: magenta contours indicate regions where H-bond acceptors on the receptor promote the affinity, while red contours indicate regions where H-bond acceptors on the receptor demote the affinity.

**Figure 4:**
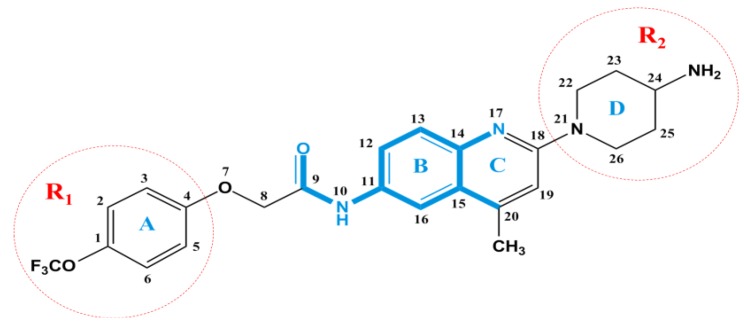
The optimal Compound **169** with common substructure of all molecules shown in bold. Two substituents R_1_ and R_2_ are denoted as red circles.

To visualize the information content of the derived CoMSIA model, the resulting coefficient × standard deviation (coeff*stddev) contour maps were analyzed. The CoMSIA contour maps, depicted in Figure 3, identify regions and their causative ligand functional groups that have crucial impact on activity and thus can be useful for ligand design [37]. The most active antagonist in the series, Compound **169** (pIC_50_ = 8.46) is exhibited as a reference structure superimposed with the contour maps to facilitate the analysis. The skeleton of Compound **169** is shown in Figure 4. The default values of favorable and unfavorable contributions ratios were set at 80% and 20%, respectively.

The PLS coefficients derived from CoMSIA steric contour plots projected onto Compound **169** are depicted in Figure 3A where green and yellow isopleths indicate the favorable and unfavorable steric interactions, respectively. It has been recognized that both green and yellow contours are observed in the region close to Ring A and Ring D where substantial modifications have been made to obtain optimized antagonists in drug design. Around the R_2_ substituent the terminal *N* points toward a small green contour region and the Ring D fits into another, which indicates the positive influence of the bulky moiety on potency in this region. This is supported by the significant loss in activity of Compound **170**, which lacks an *N*-alkyl group in Ring D compared with the two substituted analogs **168** and **169**. Moreover, there are two medium sized green contours around the distal substituent R_1_ showing a steric favored region. The two yellow contours around Ring D and the terminal N indicate that a bulky substitution would be unfavorable for this region. This can be confirmed by the loss of activity of Compound **166** with R_2_ of dimethyl piperazin compared to Compound **164** with piperazin only. The presence of a large yellow region above Ring A and 7-position implies that bulky groups are not favored here, which is consistent with the fact that Compounds **50**, **52** and **54** that possess a 7-position methyl oriented towards the yellow contour are obviously less potent than their analogs.

CoMSIA hydrophobic contours mapped onto Molecule **169** are displayed in Figure 3B. Yellow and orange contours indicate regions favorable for hydrophobic and hydrophilic groups, respectively. A distant yellow contour as well as a smaller one is located towards the substituent of Ring A at 1-positon which suggests that hydrophobic moieties at this location tend to result in a more active antagonist molecule, which may explain the increase in activity of Compound **13** substituted by a more hydrophobic trifluoromethoxyphenyl group *versus* Compound **14** with a chloromethyl. There are three orange contours above Ring A and 7,8-position signifying that hydrophilic groups are favored for activity at these positions, which is consistent with the experimental data. For example, Compound **40**, possessing a hydrophilic moiety O–CH_2_ at 7,8-position, is more potent than Compound **52** with a hydrophobic CH_3_–CH=CH instead. In addition, the region of R_2_ substituent shows the presence of an orange contour as well as another small one merged within the Ring D. This is in agreement with analogs **106**–**109** exhibiting high pIC_50_ values with terminal amine groups of hydrophilic characters.

CoMSIA H-bond donor isopleths superimposed on Compound **169** are displayed in Figure 3C. The cyan contours represent regions that prefer H-bond donors, whereas the purple contours represent regions that disfavor hydrogen bond donors. It can be seen that a cyan contour is behind Ring D at the *ortho* and *meta* positions and another minor cyan map is just under the R_2_ substituent which suggest the favored effect of H-bond donor moieties in this region. This may explain the increase in activity of Compounds **107**–**111** with R_1_ appendages of fatty amine consisting of –NH groups in 18-position. Moreover, there is also a minor cyan map near the 7- and 9-position oxygen atoms and 10-position –NH showing the favorable influence of H-bond donor groups here, which conforms to the fact that the two oxygen atoms are eager to be H-bond denoted. While the terminal *N* of R_2_ substituent in **169** points towards two big distal purple contours indicating that distal H-bond donor groups are correlated with lower pIC_50_ values of the molecules. For example, Compounds **173** and **176** are less potent than their analogs **179** and **180** substituted with –OH groups in the terminal Ring D.

CoMSIA H-bond acceptor contours mapped onto Compound **169** are shown in Figure 3D where magenta contours signify that acceptor groups at those locations on the antagonist are beneficial for activity, whereas the red enclose a region where H-bond acceptors are detrimental for the improvement of activity. Two magenta contour maps can be observed located around the terminal atom near Ring D suggesting that H-bond acceptors are preferred here, which is in accord with the fact that several derivatives possessing either hydroxyl groups or a nitrogen atom at 24-positon such as **25**, **34**, **36**, **37** exhibit higher activities than their analog **21** with methoxyl only. A red contour lies below Ring D implying the desire for an H-bond donor to improve the activity rather than an H-bond acceptor, which is consistent with our discussion in the H-bond donor part. Another medium sized red contour is flanked by Ring C covering the hydrogen atom in the 19-position indicating that the presence of a favored H-bond is not well tolerated. This could be the reason behind the reduced potency of piperazine derivative Compound **1** *versus* the quinoline derivative Compound **149**.

### 2.3. Docking Study

**Figure 5:**
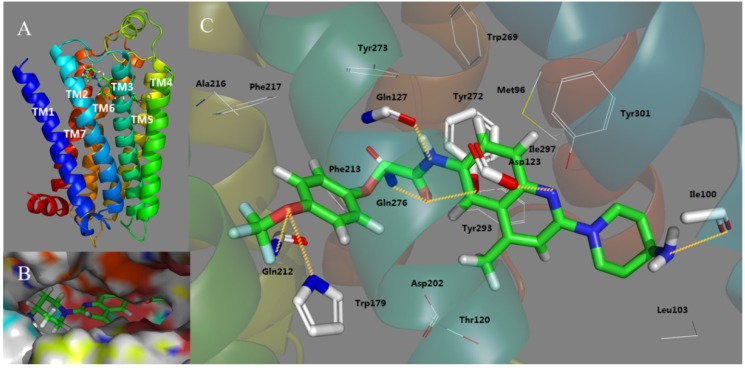
The binding mode of Compound **169** docked in MCHR1. (**A**) The overview of the docking conformation; (**B**) The cavity that the ligand fits into and the conformation of **169** in the entrance position; (**C**) The binding interactions of Compound **169** with amino acids of MCHR1. Compound **169** and interaction groups of the crucial amino acids are represented in sticks and highlighted with green and white carbons, respectively. Hydrogen bonds and salt bridges are shown as yellow dashed lines. Atoms O and N are colored red and blue, respectively.

Docking serves as an effective method to validate the stability of 3D-QSAR models previously established and explore the possible acting mechanisms between small molecule drug candidates and the target protein. For the sake of elucidating whether the MCHR1 antagonist molecules modulate the target and illustrating their interaction mechanisms as well as binding mode, docking analysis was carried out for all 181 compounds. While most attention was concentrated on protein-ligand interactions of the potent antagonist 169, the optimal conformation of which presented in Figure 5 is chosen referring to the GOLD scores.

As shown in Figure 5A, the putative binding site [38,39] of the antagonist–receptor complex was noticed to be embedded within the top half of the helical domain and located between transmembrane helices (TMs) **3**, **5**–**7**. A detailed inspection of the binding site reveals the ligand conformation and significant binding interactions demonstrated in Figure 5B,C. Compound **169** is inserted into the cavity adopting an “l” conformation with a little bending around 7-position oxygen. Almost the entire part of 169 lies along the binding pocket which is observed to be open on one side and the terminal amine (R_2_) tends to point towards the entrance of the pocket. Indeed, at the entrance location Ring D seems relatively steady, taking up most of the narrow space around it while the –NH_2_ is flexible to extend deeper into the cavity showing that introduction of a bulky substituent around 24-position and along the terminal amino disfavors and favors the binding affinity, respectively, which is in accord with the yellow and green contours near the distal substituent R_2_ depicted in Figure 3A.

As shown in Figure 5C, which clarifies the crucial amino acid residue interactions in MCHR1, the cavity that the ligand fits into is hydrophobic, and two hydrophobic subsites (listed as S1 and S2, respectively) are found to compose the binding pocket. The quinolone half of antagonist 169 is observed to be bound in S1 constituted by residues Met96, Ile100, Leu103, Tyr272, Tyr293, Ile297 and Tyr301. Sandwiched between these residues mainly characterized by aliphatics, which significantly contribute to the hydrophobic cage, the quinoline rings, B and C, and R_2_ moiety are stabilized among TM_2_, TM_3_ and TM_7_. With respect to Ring A, some aromatic residues situated near the phenyl scaffold A including Trp179, Phe213, Phe217, Trp269, Tyr272 and Tyr273 orient their chains to create an hydrophobic cage S2 which develops with a strong aromatic character for the ligand to be anchored. In addition, due to the presence of relatively bulky phenyl rings in these residues, introduction of substitutions around R_1_ position yields steric clashes which coincides well with the large yellow contour plot depicted in Figure 3A.

Aside from hydrophobic forces, key interactions include an ionic interaction (salt bridge) and six H-bonds. As is shown in Figure 5C, the carboxyl of the polar residue Asp123 is engaged in an ionic interaction with the basic quinoline nitrogen at 17-position coinciding with the experimental finding [40]. Actually, the salt bridge established between Asp123 in TM helix 3 and the charged amine in ligands represent the only experimental evidence of ligand–receptor interaction regarding the ligand pose in MCHR1 [41]. The ionic interaction formed between Asp123 and protonated amine moiety has been reported by several researchers [38,42,43]. The Asp123 interacts preferentially with the nitrogen of the central quinoline rather than the aliphatic amine [32] and plays a critical role in the binding mode stabilizing the quinoline ring in the central section of antagonists thus improving the functional activity of compounds. In addition to the essential salt bridge, six H-bond interactions were also identified, further reinforcing the affinity between the antagonist molecule and MCHR1. The carboxyl oxygen in the side chain of Gln127 is H-bonded to the 10-positon nitrogen and the branched amine of Gln276 forms a hydrogen bond to 9-position oxygen. At 1-position, the oxygen serves as an acceptor forming H-bonds donated by the nitrogen atoms in the branched chain of Gln212 and Trp179, respectively. Actually, at least one H-bond is suggested by most of the docking studies between glutamine and the polar groups, e.g., carboxyl and amine in the antagonists. These three glutamine residues mentioned above, Gln127, Gln212 and Gln276, play a critical role in our binding model. In antagonists, the presence of polar groups capable of binding Gln127 or Gln237 seems crucial for their antagonistic activity towards MCHR1 as well as their selectivity against other G protein-coupled receptors (GPCR) members [42]. An H-bond is also observed to develop between the 9-position oxygen and the side chain –OH of Tyr272 which plays a role of hydrogen bond donor. These H-bonds contribute to the stabilization of the R_1_ side of the ligand together with the aromatic S1 previously mentioned. Moreover, the ligand is further anchored within the binding pocket with an H-bond interaction formed by the backbone hydroxy group of Ile100 and the 21-position nitrogen acting as a hydrogen bond acceptor, which facilitates the stabilization of the other side of the ligand.

All in all, the participation of hydrophobic and ionic interactions as well as H-bonds results in the approximately linear conformation of the ligand anchored in MCHR1. These interactions along with the 3D-QSAR models generated presently may provide us with useful information for designing more selective and potent MCHR1 antagonists in the future.

### 2.4. Molecular Dynamics (MD) Analysis

Obviously, the clarification of ligand binding mechanisms is an essential step. A construction of the protein model feeling its natural environment is needed and MD simulation is one of the best methods for such a refinement [44]. Furthermore, unlike molecule docking that neglects the protein flexibility, MD simulation seems more reliable with a view to the conformational flexibility and atomic-level dynamics of proteins computationally exploring the structure and dynamics of biological macromolecules [26]. Thus, MD simulation was adopted to assess the reliability of the interaction model system and estimate the binding affinity of the ligand. Here, we conduct MD simulations in two different environments, with the receptor in water and embedded in the lipid bilayer, respectively. The contrast of the MD processes performed in different situations may help test whether the inclusion of the receptor within lipid bilayer affects the results.

#### 2.4.1. Receptor in Water

**Figure 6:**
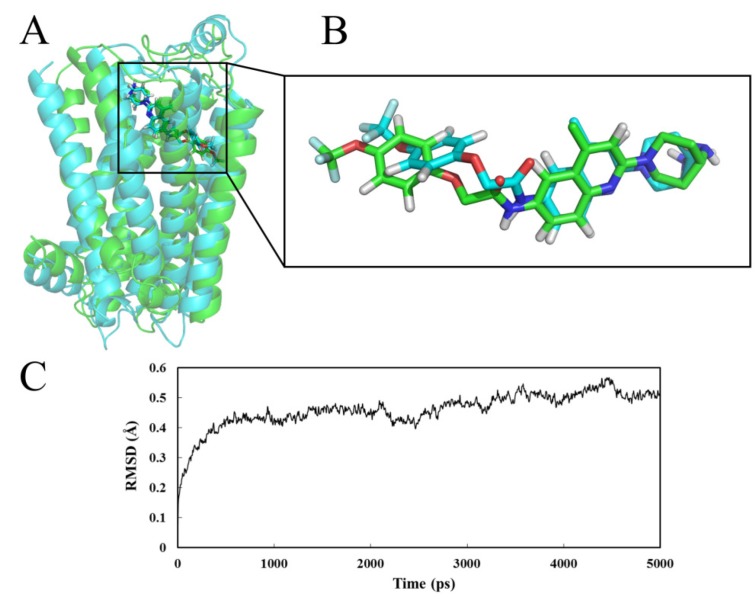
(**A**) View of the superimposed backbone atoms of the average structure for the last 1 ns of the molecular dynamics (MD) simulation (green) and the initial structure (cyan) for the Compound **169**–MCHR1 complex; (**B**) The initial and the final average structures of Compound **169** shown in cyan and green sticks; (**C**) Plot of the root-mean-square deviation (RMSD) of docked complex versus MD simulation time in the MD-simulated structures.

A 5ns simulation was performed with the docked complex of MCHR1 as starting molecular structure, and Figure 6 shows the dynamical image of the conformational alterations taking place in aqueous solution. Figures 6A,B illustrate the average structure of the last 1 ns during the MD process (as shown in green) superposed by the initial docked structure (as shown in cyan), with the initial and the final average structures of Compound **169** shown in cyan and green sticks, respectively. It is worth mentioning that the adoption of the average structure in the last 1 ns shows more reliance compared to the use of a single crystal structure [45]. To explore the dynamic stability of the complex and ensure the rationality of the sampling method, the root-mean-square deviation (RMSD) as a geometric measure of conformational diversity was monitored regarding the initial structure, ranging from 0.20 to 0.56 Å, as depicted in Figure 6C. The plot demonstrates that the RMSD of the system reaches a converged stage after 3.0 ns, retaining about 0.50 Å throughout the simulation indicating that the MD trajectory is well equilibrated and behaves rather stable in the system for docked complex structure.

**Figure 7:**
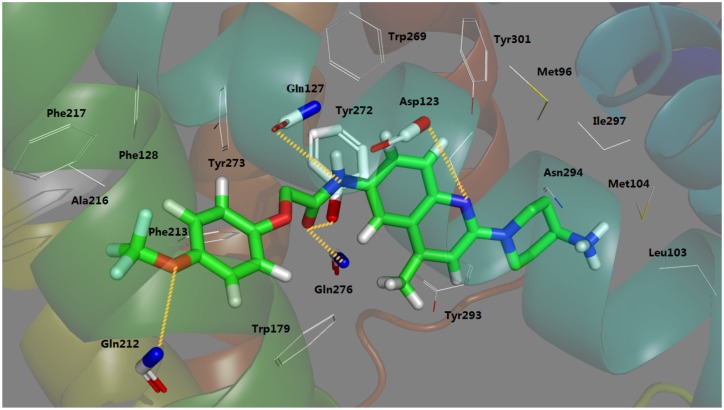
Plot of the in-water MD-simulated structures of the binding site. Compound **169** and interaction groups of the crucial amino acids are represented in sticks and highlighted with green and white carbons, respectively. Hydrogen bonds and salt bridges are shown as yellow dashed lines. Atoms O and N are colored red and blue, respectively.

As is observed in Figure 6A, the ligand **169** in docking and MD simulations owns the same binding site without any significant changes in the structural conformation, which verifies the rationality of the docking model. Yet the limited conformational variation that Ring A of the MD average structure extends more straightly into the putative pocket rather than rotates at some angle as the docking ligand does, may not be over-looked. In view of the possible interaction changes between Molecule **169** and MCHR1 resulting from the mobility and variety of the complex system compared to the docking analysis, the binding mode derived from MD simulation was also investigated in terms of hydrophobic contacts, ionic bond and H-bond interactions as depicted in Figure 7.

As anticipated, these interactions fit well with those revealed in the docking simulation. The quinoline and R_2_ part of Compound **169** is anchored in a hydrophobic cage constituted by residues Leu103, Met104, Tyr272, Tyr293, Ile297 and Tyr301. Another hydrophobic aromatic region constituted by Phe128, Trp179, Phe213, Phe217, Tyr272 and Tyr273 is centered around Ring A. Apparently both parts coincide with hydrophobic subsites S1 and S2 in the docking study. In addition, the crucial ionic bond acting between Asp123 and the basic nitrogen at 17-position is predictably retained in the MD result. Furthermore, with respect to important hydrogen bonds, four H-bond interactions described in detail in the preceding docking model also emerged in this MD binding system, *i.e.*, the amino group of Gln276 together with the hydroxy moiety of Tyr272 serves as H-bond donor affecting the oxygen atom at 7-position; the carboxyl oxygen of Gln127 is hydrogen bonded to the 10-position nitrogen; Gln212 forms an H-bond to the terminal 1-position oxygen acting as a donor. In spite of the above reproductions of binding interactions exhibited in the MD result further supporting the docking model, it is worth mentioning that subtle differences arise in the putative pocket. Owing to the approximately linear conformation of Compound **169** during MD simulation, changes that occurred in H-bond formations are observed. The hydrogen bond obtained between Trp179 and the terminal oxygen in previous docking analysis is broken, so is the H-bond between Ile100 and the terminal –NH_2_. The absence of these two stretching forces may lead to the straighter conformation of the ligand in the MD result. All in all, despite the slight discrepancy presented in the result of MD simulation, the docking model shows a rationality suggesting helpfulness and reliability for modification and design of potent MCHR1 antagonists.

#### 2.4.2. Receptor in Lipid Bilayer

**Figure 8:**
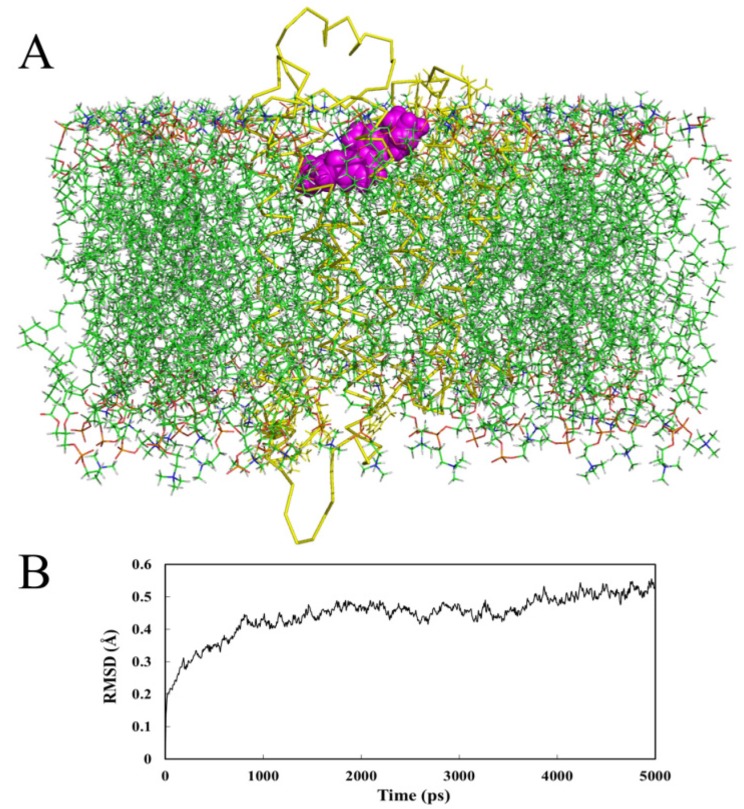
(**A**) The receptor with docked ligand within the lipid bilayer after 5 ns of MD simulation. Protein is shown as ribbons. Ligand is shown as spheres. Lipid molecules are shown as lines; (**B**) Plot of RMSD of docked complex *versus* the MD simulation time in the MD-simulated structures.

A simulation with the Ligand **169** embedded in a lipid bilayer environment was performed. The snapshot of the ligand-protein complex and the plot of RMSD are depicted in Figure 8. As shown in Figure 8A, Compound **169** stays almost at the same position as in the docking analysis. In Figure 8B, the plot demonstrates that the backbone RMSD of the system remains constant at approximately 4.8 Å after 3.8 ns, which shows a stable MD trajectory as well.

**Figure 9:**
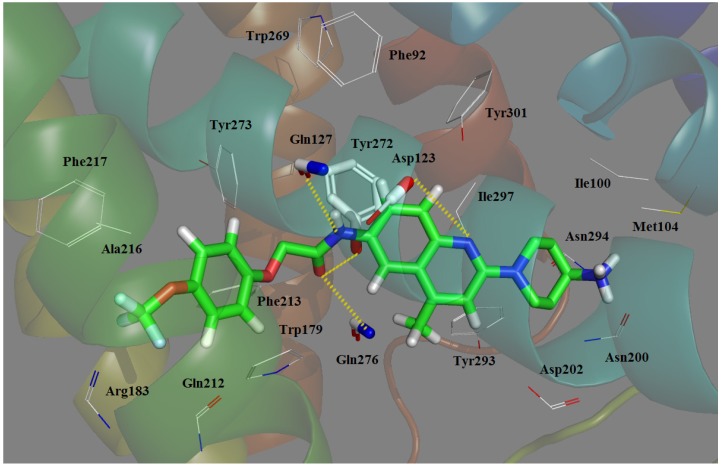
Plot of the in-lipid MD-simulated structures of the binding site. Compound **169** and interaction groups of the crucial amino acids are represented in sticks and highlighted with green and white carbons, respectively. Hydrogen bonds and salt bridge are shown as yellow dashed lines. Atoms O and N are colored with red and blue, respectively.

The binding mode of the ligand after 5 ns MD simulation in lipid bilayer is displayed in Figure 9. Compound **169** is situated in the same hydrophobic binding site as mentioned above. The two subsites, S1 and S2, are also reserved. Besides hydrophobic effect, salt bridge and H-bonds are listed as follows: Asp123 forms a salt bridge to the quinoline nitrogen; Gln127 is H-bonded to the 10-position nitrogen as a H-bond donor; Tyr272 and Gln276 form H-bonds to the 9-position oxygen as H-bond donors. These interactions comform well with the preceding docking and in-water MD simulations. All in all, in both cases of the MD analyses, the ligand is stable within the active site and both MD results agree with the docking analysis.

### 2.5. Docking Comparison

The application of the docking method has been accompanied with the development of MCHR1 antagonists ever since 2004. Several docking studies on this receptor have been reported contributing to the exploitation and optimization of potent small antagonist molecules. In view of the relatively extensive employment of docking analysis in this field, a comparison exploring the resemblances and discrepancies of our and other resultant docking models between was conducted focusing on the binding mode and interaction features. Some crucial information of this research is listed in Table 2.

**Figure 10:**
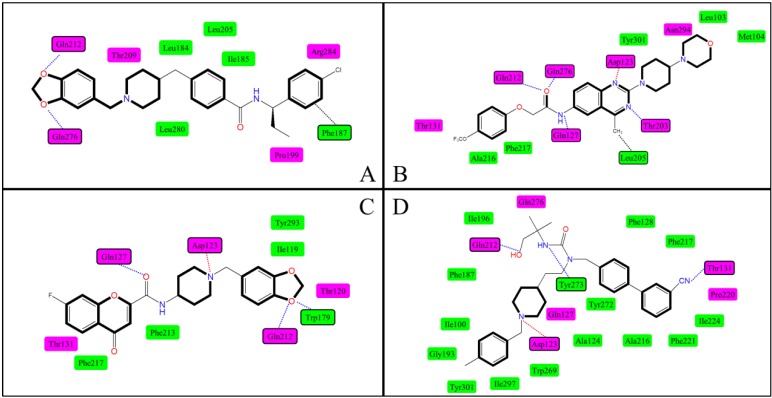
Binding modes of MCHR1selected from docking results performed by others. (**A**) The interaction map of P2 obtained by Abu-Hammad *et al*. [49]; (**B**–**D**) The interaction maps of P1 obtained by Sasmal *et al*. [29], Helal *et al*. [39] and Cirauqui *et al*. [43], respectively. H-bonds are shown as blue dashed lines, ionic interactions in red and van der Waals force in black. The green squares denote the hydrophobic residues and the pink signify the poly.

**Figure 11:**
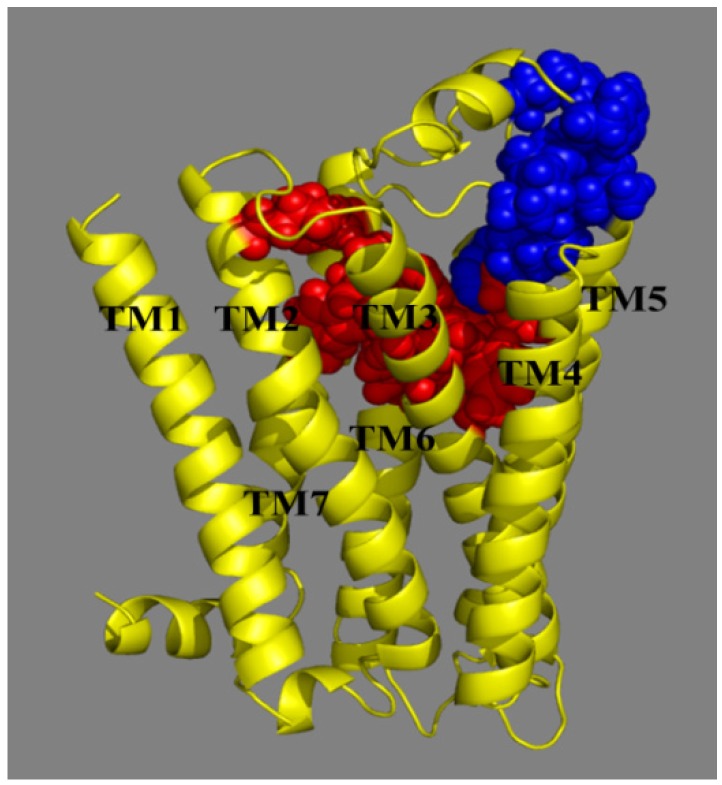
Two binding sites of MCHR1. P1 are depicted in red located between TMs 3, 5–7. P2 is depicted in blue between TMs 3–5.

To the best of our knowledge so far [29,32,38,39,42,43,46,47,48,49,50,51], two MCHR1 binding pockets (listed as P1 and P2, respectively) have been proposed. P1 represents the conventional binding cavity that almost all docking researches have referred to where a typical interaction of salt bridge is found experimentally formed by Asp123, and simultaneously H-bond or hydrophobic regions may also be embodied. Crucial residues of P1 are composed of Phe213, Phe217, Gln212, Tyr272, Tyr273, Tyr293, Tyr301, Gln276, Gln127 besides Asp123. An elaborated discussion of the binding mode of P1 is provided later. With regard to P2, it was only introduced by Abu-Hammad *et al*. [49] in 2009. Unlike those in P1, binding forces in P2 contain van der Waals stacking instead of ionic interaction in addition to the hydrophobic effect and H-bond. As noticed in the arrangement of residues around P2 depicted in Figure 10A, crucial amino acids consist of Leu184, Ile185, Phe187, Pro199, Leu205, Thr209, Gln212, Leu280, Arg284 and Gln276, from which we infer that the location of P2 borders to that of P1 with common residues Gln212 and Gln276. These two residues participate in the interaction with MCHR1 antagonists as well forming hydrogen bonds. A van der Waals stacking between the phenyl moiety and Phe187 was also observed. The visualized positions of both cavities are illustrated in Figure 11.

**Table 2:** Important information of docking studies performed by other researchers.

No.	Researchers	Template	Binding Site	Binding Interactions	Crucial Residues
1	Clark *et al*. [42]	Bovine rhodopsin	P1	Ionic interaction, H-bond, hydrophobic effect	Asp123, Gln276
2	Tavares *et al*. [38]	Bovine rhodopsin	P1	Ionic interaction, H-bond	Asp123, Tyr273
3	Witty *et al*. [46]	Bovine rhodopsin	P1	Unclear	Asp123
4	Giordanetto *et al*. [47]	1U19 ^a^	P1	Ionic interaction, H-bond	Asp123, Gln127
5	Cavasatto *et al*. [48]	1L9H ^a^	P1	π–π stacking, H-bond	Asp123, Gln127 Trp269, Ile297, Gly300, Tyr301
6	Abu-Hammad *et al*. [49]	1U19 ^a^	P2	Van der Waals stacking, H-bond, hydrophobic effect	Gln212, Gln276 Phe187
7	Sasmal *et al*. [50]	2RH1 ^a^, 2VT4 ^a^	P1	Ionic interaction, H-bond, hydrophobic effect	Asp123, Gln127, Gln212, Gln268, Asn255
8	Ulven *et al*. [32]	1F88 ^a^	P1	Ionic interaction, H-bond, hydrophobic effect	Asp123, Gln212
9	Sasmal *et al*. [29]	2RH1 ^a^	P1	Ionic interaction, H-bond	Asp123, Gln127, Gln212, Gln276, Thr164
10	Helal *et al*. [39]	1U19 ^a^	P1	Ionic interaction, H-bond, hydrophobic effect	Asp123, Gln127, Gln212, Gln276, Trp179, Thr131
11	Cirauqui *et al*. [43]	1U19 ^a^	P1	Ionic interaction, H-bond, hydrophobic effect	Asp123, Gln212, Gln276,Tyr273, Thr131
12	Kamata *et al*. [51]	1F88 ^a^	P1	H-bond, hydrophobic effect	Gln127, Asn294

^a^ PDB ID of the template.

Apparently, in light of the amino acid profile settled around the small ligand, the cavity that Antagonist **169** is docked into accords well with the customary P1. In addition, with regard to the majority of docking results that show binding effect in P1, the docking modes can be sorted into three classes according to various configurations of the small ligands. We use these three modes here for further analysis and comparee them with our own binding model.

Mode I. In the first place, a model resulting from the work of Sasmal *et al*. (listed as No. 9) is presented here. The best hit, Compound **42**, in their work, which is linear with a terminal nitrogen, was subjected to a full flexible ligand docking study with the X-ray structure of β2-adrenergic receptor (PDB:2RH1) utilized as template in homology modeling. With a slight curving at the 7-position oxygen, Compound **42** adopts an approximately linear conformation according to their resultant docking pose. The planar binding interactions between the protein and **42** are displayed in Figure 10B. One of the quinazoline N atoms participates in the salt bridge with Asp123, however, the other develops an H-bond to Thr203. Three other crucial H-bonds were also identified for Gln127, Gln212 and Gln276. Besides, a van der Waals force is noticed between the 4-methyl of quinazoline and Leu205.

In Mode I, a bicyclic amine system is generally involved in the central section of the ligand, which plays an important role in the stabilization of ligand–MCHR1 complex. H-bonds formed with the interchain O and N atoms, hydrophobic pockets around both sides of the ligand and a van der Waals stacking may also participate in the binding mode. Similar docking results that pertain to Mode I are able to be observed in No. 1, 2, 7, 8, 12 as listed in Table 2 as well.

When comparing our docking results with Mode I, *i.e.*, through the comparison between molecules **169** and **42**, it is found that their interaction mechanisms are quite similar to each other. Despite their skeleton discrepancies that **42** replaces the quinoline rings with a quinazoline system as well as the terminal -NH_2_ with cyclic 2-pyrrolidinone, major forces are mostly retained, especially the ionic and hydrogen interactions formed to Asp123, Gln127, Gln212 and Gln276. While it is worth pointing out that two of the H-bonds have transformed in terms of acceptor atoms in the docking mode of Compound **42**, *i.e.* both the branched amino of Gln276 and Gln212 turn to form hydrogen bonds with the 9-position oxygen, rather than interact with that at 1- or 7-position. This may be induced by the steric clashes of plentiful phenyl groups around R1, which bring about varying degrees of bending at 7-, 8- or 9-position followed by a shift of the ligand, which may also explain the turning-up of the H-bond between Tyr233 and the 9-position oxygen. Moreover, due to the presence of quinazoline instead of quinoline, Thr203 is found to be hydrogen bonded to the added quinazoline nitrogen.

Mode II. New insights into the binding mode of MCHR1 antagonists have been disclosed by Helal *et al*. (No. 10) and Compound **4** in their work exemplified as a chemotype linear with central basic nitrogen was studied in a docking simulation. The homology model was constructed with the crystal structure of bovine rhodopsin (PDB: 1U19) selected as a template. Two possible hydrophobic pockets (P1, P2) were noticed around the basic nitrogen for this linear type and the docking model with P1 is cited here for further investigation due to the similarity of H-bonding properties within this region between **4** and **169**. As illustrated in Figure 10C, the proposed binding mode of Antagonist **4** encompasses a salt bridge with Asp123 and an H-bond to Gln127. Unlike the flexible Compound **169** that possesses links to Gln212 and Gln276, more rigid molecules, like 4, have difficulty with these interactions. It is notable that, because of the presence of suitable H-bond acceptor oxygen atoms, the hydrophobic moiety Rings A and B curve to fill in this P1 pocket forming hydrogen bonds to Gln212 and Trp179, which coincides well with our docking mode where the 1-position oxygen serves as an acceptor forming H-bonds to both Gln212 and Trp179.

The binding interactions in Mode II resemble those in Mode I due to the configuration similarity of the ligands in both modes. The main structure discrepancy is that the Mode II ligands are short of terminal basic N compared to the small antagonist molecules in Mode I and this may cause instability and curving at the end of the chain, which renders the terminal part of the ligand fitted in different possible hydrophobic subsites. Similar docking results may be noticed according to No. 4 in Table 2.

Mode III. Cirauqui *et al*. (No. 12) conducted a docking process with a homology model of MCHR1 based on the crystal structure of bovine rhodopsin (PDB: 1U19). Compound **2t**, a branched best hit in their work, was docked in the receptor model. The proposed binding mode of their resultant receptor/ligand complex is illustrated in Figure 10D. A salt bridge is formed between Asp123 and the basic amine of **2t**. Three generated H-bonds are also present: Gln212 bond to the hydroxyl group; Tyr273 interact with the urea hydrogen atom; Thr131 is hydrogen bonded to the cyano group. Evidently, the crucial amino acids Asp123 and Gln212 are well retained in our work. Rings A and B of Compound 2t, the *p*-toluyl and basic amine moiety, are located in the same hydrophobic region where the R_1_ part of Compound **169** binds between residues Met96, Ile100, Ile297, Tyr301 and Trp269 in our study. As for Rings C and D, the biphenyl group, they are predicted to be bound in a second hydrophobic region with an aromatic trait. Part of this aromatic pocket is overlapped with ours in view of Ala216, Phe217, Tyr272 and Tyr273 while the rest are situated adjacently surrounded by residues Phe128, Pro220 and Phe221, which may come out on account of the branched configuration of **2t** with a larger bending angle.

Mode III shows the binding mode of antagonists with branched configuration. Binding-active O and N atoms plays a part in forming H-bonds or ionic interactions at different branched chains located in certain hydrophobic pockets. A similar situation is also noticed in the docking results of No. 5 in Table 2.

To summarize, three binding modes of MCHR1 antagonists have been achieved in P1 by other researchers regarding to various configurations of the ligands. Our docking results obey the binding characteristics of Mode I where the ligand with terminal nitrogen adopts an approximately linear conformation stabilized by salt bridge, H-bond, hydrophobic effect, *etc.* The docking comparison of Compounds **169** and **42**, which are similar in constitution, shows that they complement and validate each other. Modes II and III represent the docking models of ligands which are linear with a central basic nitrogen and branched, respectively. In spite of the discrepancies shown between these three modes, there is some generality in binding interactions: a salt bridge with Asp123, several H-bonds with glutamines and at least one hydrophobic pocket. These may help provide insights into the binding of different types of MCHR1 antagonists.

## 3. Experimental Section

All 3D-QSAR molecular modeling processes were executed using the program SYBYL 6.9 (Tripos Associates, St. Louis, MO, USA), running on a Linux environment. All the structures were energy-minimized applying the Tripos force field [52] with a distance-dependent dielectric and the Powell conjugate gradient algorithm with a convergence, and partial atomic charges were figured up by the Gasteiger–Hückel method [53].

### 3.1. Biological Activities and Dataset Construction

The dataset in our study consists of a series of 181 quinoline/quinazoline derivatives published with a wide scope of antagonistic activities (IC_50_) against MCHR1. The *in vitro* IC_50_ values in nm were transformed into corresponding pIC_50_ (−logIC_50_) values ranging from 5.02 to 8.46, which were used as dependent variables in model constructions in the following *in silico* analysis. In an approximate ratio of 2:1, the entire dataset was separated into two groups randomly, generating a training set of 121 compounds structuring the model and a test set of 60 molecules evaluating the validation of the model, respectively. Both sets were selected on the basis that molecules in the test set should appropriately represent the diversity of the structure and the distribution activity of those in the training one. All the structures and biological activities of the data set are displayed in Supplementary Table S1–S13.

### 3.2. Conformational Optimization and Alignment

**Figure 12:**
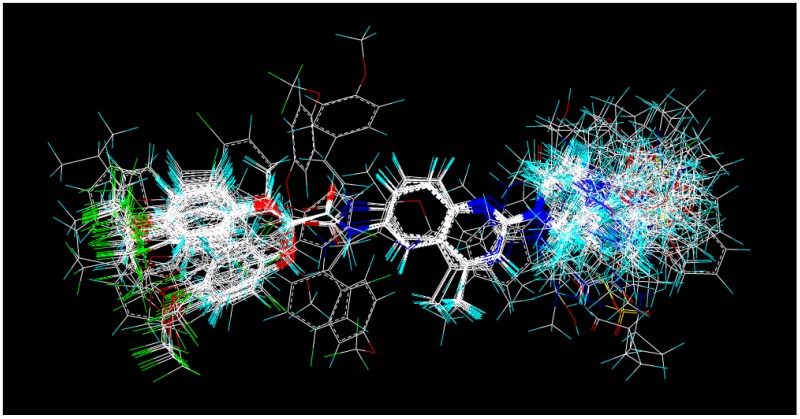
Ligand-based alignment of all compounds.

As one step for the successful development of CoMFA and CoMSIA models, molecular alignment is of great significance [54]. In order to build the most efficient and reliable model, a ligand-based alignment method was applied. In this approach, the most effective Compound **169** was chosen as the template to fit the rest of the compounds with the common scaffold using the “align database” procedure in SYBYL6.9 (Tripos, St. Louis, MO, USA). The resultant model is shown in Figure 12.

### 3.3. Comparative Molecular Field Analysis (CoMFA) and CoMSIA Studies

CoMFA and CoMSIA, considered to be effective methodologies in 3D-QSAR analysis, are widely applied. Herein, we used those two techniques to explore the relationship between MCHR1 antagonist activities and 3D structural features and predict the influence the interactive fields may exert on the activity. Both CoMFA and CoMSIA studies were performed in SYBYL 6.9 with the default parameters.

To build the CoMFA model, two descriptors were calculated: steric (S) and electrostatic (E) field energies, obtained on the basis of Lennard-Jones potential and Coulombic potential, respectively [55]. The activity of compounds is relevant to their steric and electrostatic interaction energies [56]. To derive CoMFA descriptors fields, a 3D cubic lattice with grid spacing of 2.00 Å beyond the aligned molecules in all directions was created [57]. Steric and electrostatic interactions were calculated utilizing a hybridized sp^3^ carbon probe atom with a van der Waals radius of 1.52 Å and a charge of +1 at each lattice point. Energy truncation value of 30 kcal/mol was set for both the steric and electrostatic fields with a distance-dependent dielectric constant.

For the construction of the CoMSIA model, besides steric (S) and electrostatic (E), another three descriptors, namely the hydrophobic (H), H-Bond donor (D) and H-bond acceptor (A) were involved. The five similarity indices were generated using the same lattice boxes as in CoMFA calculations with grid spacing of 2.00 Å and employing the sp^3^ atom with a radius of 1.00 Å, charge +1.0, D and A properties of +1.0. CoMSIA similarity indices (*A*_F_) for molecule *j* with atoms *i* at a grid point *q* were calculated using the following equation:

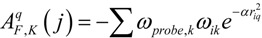
(1)
where *k* represents the following physicochemical properties: steric, electrostatic, hydrophobic, H-bond donor and H-bond acceptor. A Gaussian type distance dependence was used between grid point *q* and each atom *i* of the molecule. The default value of 0.3 was used as the attenuation factor α.

Considering the greater robustness that CoMSIA methodology possesses compared with CoMFA, CoMSIA methodology is supposed to be more efficient. In addition, CoMSIA studies have advantages of being carried out without a process of energy cut-off and obtaining more contour maps for better analysis [26].

### 3.4. Partial Least Squares (PLS) Analysis and Validation of Quantitative Structure–Activity (QSAR) Models

To build statistically significant 3D-QSAR models, the models were quantified using partial least squares (PLS) regression analysis. The PLS algorithm was adopted to establish correlations between the CoMFA/CoMSIA descriptors and MCHR1 pIC_50_ values, as independent and dependent variables, respectively. The PLS was divided into two stages. In the first stage, cross-validation was performed using the leave one out (LOO) methodology to evaluate the reliability of the models, which determined the optimum number of components (OPN) to be further applied to derive final regression model, the conventional correlation coefficient (*Q*^2^) and the standard predicted errors (SEP).The *Q*^2^ was calculated with equation:

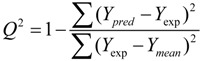
(2)
where *Y*_pred_, *Y*_exp_ and *Y*_mean_ are the values for the predicted activity, experimental activity and mean activity, respectively.

In the next stage, non-cross-validation analysis was developed using the resulting OPN to generate the final PLS regression models for CoMSIA [58] with the Pearson coefficient (*R*^2^_ncv_), standard error of estimate (SEE) and F test ratio calculated subsequently. In addition, by means of predicting the activity of the test set, the external predictive correlation coefficient (*R*^2^_pred_) was obtained to evaluate the external predictive ability of the CoMSIA model [59]. Finally, the CoMFA/CoMSIA results were graphically displayed by contour maps for further analysis.

### 3.5. Homology Modeling

Due to the absence of available experimentally determined atomic structure, a homology modeling method was applied. The protein sequence of human MCHR1 with the entry number Q99705 was retrieved from the universal protein resource (UniProtKB) database [60] in fasta format. The recently reported X-ray structure of β-adrenergic receptor (PDB: 2RH1) [61] taken from RCSB Protein Data Bank (Brookhaven National Laboratory, New York, NY, USA) [29] was selected as the homology modeling template due to its high sequence similarity to MCHR1. Both the MCHR1 sequence and structure were submitted to the Swiss-Model Workspace [62] to obtain the theoretical structure of MCHR1 protein target automatically.

### 3.6. Docking Simulations

For the sake of identifying the bioactive conformation and revealing the binding mechanism between MCHR1 and its antagonists, we employed docking simulations with the GOLD (Genetic Optimization of Ligand Docking) version 5.1 program, based on the genetic algorithm [63]. The ligands were docked into the ligand-binding domain (LBD) of MCHR1 to explore the full range of ligand conformational flexibility with protein partial flexibility [64]. Prior to docking, protein models were first modified with polar hydrogen atoms added to the protein structure. The binding site was defined by inputting the serial number of an atom around which the residues in space within 10 Å were involved. Each of all 181 molecules was docked into the potential binding site generating 20 possible conformations. All conformations were evaluated with GOLD fitness score, which is calculated considering contributions of H-bond and van der Waals interactions between MCHR1 and the ligand along with intramolecular H-bonds and strains of the ligand, which indicate the veracity of the conformations [65]. In the docking process, the protein structure stayed rigid while the structures of ligands were flexible.

### 3.7. MD Simulations

Utilization of MD simulation has been a widespread method in calculating physical movement of molecules as a function of time and it assesses the reliability of an interaction model system through simulations by comparison with experiments in a genuine situation [66]. To get the realistic binding affinity of the docked complex with the most potent Compound **169** and examine the stability of the docking solution, exhaustive MD simulations were performed with GROMACS software package [67]. The topology file of molecules for the protein ligand was generated in PRODRG 2.5 [68]. The complex structure was immersed in a cubic periodic box with a side length of 1 Å and at least 10 Å apart from any point in the protein. All atoms belonging to protein complexes and water were placed randomly in the box to guarantee electroneutrality of the system. The remaining space in the simulation system was filled with Simple Point Charge (SPC) water [69].

Then, the entire system was energy-minimized without constraints using the steepest descent algorithm and then equilibrated via a 500 ps MD simulations at 300 K before a 5 ns simulation was carried out with a time step of 2 fs.

In the simulation process mentioned above, the calculation was conducted using the GROMOS96 force field [70] combined with a periodic boundary condition that used the particle mesh Ewald (PME) method [71] and an NPT ensemble at 300 K that used a normal pressure and temperature [72]. The Berendsen thermostat method was used to keep the temperature constant. The value of the isothermal compressibility was set to 4.5 × 10^−5^ bar^−1^ and the pressure retained at 1 bar using the Parrinello–Rahman scheme [73]. Criterion distances for calculating the Coulomb and van der Waals interactions were assigned to 1.0 and 1.4 nm, respectively.

MD was also performed within an explicit dioleoylphosphatidylcholine (DOPC) lipid bilayer. This is more realistic since MCHR1 is a member of GPCR having seven transmembrane structures. The lipid bilayer system was built applying the Charmm input generator, graphical interface [74,75]. The same MD process was performed as above.

## 4. Conclusions

**Figure 13:**
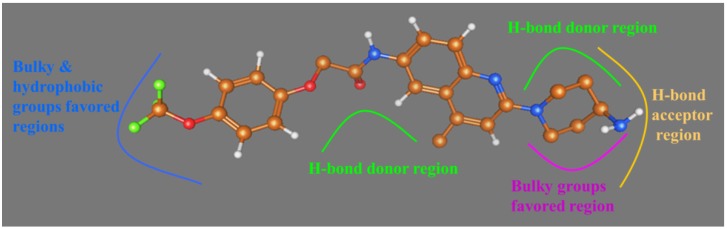
The interaction features of Compound **169** impacting the antagonistic activity obtained from our present work.

In the present study, the up-to-date largest dataset of 181 quinoline/quinazoline derivatives as potential MCHR1 antagonists was subjected to a comprehensive *in silico* study with integration of 3D-QSAR analysis, homology modeling, docking and MD simulations. Both CoMFA and CoMSIA methods were employed for the construction of ligand-based and receptor-based 3D-QSAR models and the optimal predictable CoMSIA model exhibited significant validity with *Q*^2^ = 0.509, *R*^2^_ncv_ = 0.841 and *R*^2^_pred_ = 0.745. In addition, an intuitive insight into the structural determinants of quinoline/quinazoline derivatives was performed based on the corresponding contour maps and the crucial structural traits are depicted in Figure 13. The recently reported X-ray crystal structure of β-adrenergic receptor (PDB:2RH1) was utilized as template in homology modeling due to its high sequence similarity to MCHR1. Moreover, the comparison of docking and MD analyses along with the CoMSIA results show a preferable identity, suggesting reliability and robustness of the model. To sum up, our main findings:

(1) The most potent Compound **169** fits into the conventional binding pocket P1 and follows the binding mode of Mode I with the ligand owning a central basic nitrogen taking an approximately linear configuration.
(2) Two hydrophobic subsites S1 and S2 are observed to compose P1 of MCHR1.
(3) Antagonist **169** forms a conventional interaction of salt bridge with Asp123 and six H-bonds with Gln127, Gln212, Gln276, Tyr272, Trp179 and Ile100. To the best of our knowledge, this is the first time the role of Tyr272 and Ile100 in hydrogen bonds is noticed in the ligand–MCHR1 complex.
(4) In general, a salt bridge with Asp123, several H-bonds with glutamine and at least one hydrophobic pocket are usually involved in the binding of MCHR1 antagonists.
(5) MD analysis processed within a lipid bilayer environment shows similar results to those performed in water.

All in all, we anticipate that the knowledge gained from our models will facilitate the design and optimization of novel MCHR1 antagonists as promising anti-obesity agents.

## Supplementary Materials

Supplementary Tables can be found at http://www.mdpi.com/1422-0067/15/9/15475/s1.

## Supplementary Files

Supplementary File 1

## References

[1] Kopelman P.G. (2002). Obesity as a medical problem. Nature.

[2] Ogden C.L., Carroll M.D., Curtin L.R., McDowell M.A., Tabak C.J., Flegal K.M. (2006). Prevalence of overweight and obesity in the United States, 1999–2004. J. Am. Med. Assoc..

[3] Bray G.A., Bellanger T. (2006). Epidemiology, trends, and morbidities of obesity and the metabolic syndrome. Endocrine.

[4] Luthin D.R. (2007). Anti-obesity effects of small molecule melanin-concentrating hormone receptor1 (MCHR1) antagonists. Life Sci..

[5] Hofbauer K.G., Nicholson J.R., Boss O. (2007). The obesity epidemic: Current and future pharmacological treatments. Annu. Rev. Pharmacol. Toxicol..

[6] Kawauchi H., Kawazoe I., Tsubokawa M., Kishida M., Baker B. (1983). Characterization of melanin-concentrating hormone in chum salmon pituitaries. Nature.

[7] Vaughan J.M., Fischer W.H., Hoeger C., River J., Vale W. (1989). Characterization of melanin-concentrating hormone from rat hypothalamus. Endocrinology.

[8] Presse F., Nahon J.L., Fischer W.H., Vale W. (1990). Structure of the human melanin concentrating hormone mRNA. Mol. Endocrinol..

[9] Elias C.F., Saper C.B., Maratos-Flier E., Tritos N.A., Lee C., Kelly J., Tatro J.B., Hoffman G.E., Ollmann M.M., Barsh G.S. (1998). Chemically defined projections linking the mediobasal hypothalamus and the lateral hypothalamic area. J. Comp. Neurol..

[10] Rossi M., Beak S.A., Choi S.-J., Small C.J., Morgan D.G.A., Ghatei M.A., Smith D.M., Bloom S.R. (1999). Investigation of the feeding effects of melanin concentrating hormone on food intake-action independent of galanin and the melanocortin receptors. Brain Res..

[11] Gomori A., Ishihara A., Ito M., Mashiko S., Matsushita H., Yumoto M., Ito M., Tanaka T., Tokita S., Moriya M. (2003). Chronic intracerebroventricular infusion of MCH causes obesity in mice. Am. J. Physiol. Endocrinol. Metab..

[12] Shimazaki T., Yoshimizu T., Chaki S. (2006). Melanin-concentrating hormone MCH1 receptor antagonists. CNS Drugs.

[13] Ludwig D.S., Mountjoy K.G., Tatro J.B., Gillette J.A., Frederich R.C., Flier J.S., Maratos-Flier E. (1998). Melanin-concentrating hormone: A functional melanocortin antagonist in the hypothalamus. Am. J. Physiol. Endocrinol. Metab..

[14] Ludwig D.S., Tritos N.A., Mastaitis J.W., Kulkarni R., Kokkotou E., Elmquist J., Lowell B., Flier J.S., Maratos-Flier E.J. (2001). Melanin-concentrating hormone overexpression in transgenic mice leads to obesity and insulin resistance. J. Clin. Investig..

[15] Shimada M., Tritos N.A., Lowell B.B., Flier J.S., Maratos-Flier E. (1998). Mice lacking melanin-concentrating hormone are hypophagic and lean. Nature.

[16] Tana C.P., Sanob H., Iwaasab H., Pana J., Sailera A.W., Hreniuka D.L., Feighnera S.D., Palyhaa O.C., Ponga S.-S., Figueroac D.J. (2002). Melanin-concentrating hormone receptor subtypes 1 and 2: Species-specific gene expression. Genomics.

[17] Chambers J., Ames R.S., Bergsma D., Muir A., Fitzgerald L.R., Hervieu G., Dytko G.M., Foley J.J., Martin J., Liu W.-S. (1999). Melanin-concentrating hormone is the cognate ligand for the orphan G-protein-coupled receptor SLC-1. Nature.

[18] Chaki S., Funakoshi T., Hirota-Okuno S., Nishiguchi M., Shimazaki T., Iijima M., Grottick A.J., Kanuma K., Omodera K., Sekiguchi Y. (2005). Anxiolytic- and antidepressant-like profile of ATC0065 and ATC0175: Nonpeptidic and orally active melanin-concentrating hormone receptor 1 antagonists. J. Pharmacol. Exp. Ther..

[19] Johansson A. (2011). Recent progress in the discovery of melanin-concentrating hormone 1-receptor antagonists. Expert Opin. Ther. Pat..

[20] Takekawaa S., Asamia A., Ishiharab Y., Terauchib J., Katob K., Shimomuraa Y., Moria M., Murakoshic H., Katoc K., Suzuki N. (2002). T-226296: A novel, orally active and selective melanin-concentrating hormone receptor antagonist. Eur. J. Pharmacol..

[21] Borowsky B., Durkin M.M., Ogozalek K., Marzabadi M.R., DeLeon J., Heurich R., Lichtblau H., Shaposhnik Z., Daniewska I., Blackburn T.P. (2002). Antidepressant, anxiolytic and anorectic effects of a melanin-concentrating hormone-1 receptor antagonist. Nat. Med..

[22] Högberg T., Frimurer T.M., Sasmal P.K. (2012). Melanin concentrating hormone receptor 1 (MCHR1) antagonists—Still a viable approach for obesity treatment?. Bioorg. Med. Chem. Lett..

[23] Me´ndez-Andino J.L., Wos J.A. (2007). MCH-R1 antagonists: What is keeping most research programs away from the clinic?. Drug Discov. Today.

[24] Valentino M.A., Lin J.E., Waldman S.A. (2010). Central and peripheral molecular targets for antiobesity pharmacotherapy. Clin. Pharm. Ther..

[25] MacNeil N.J. (2013). The role of melanin-concentrating hormone and its receptors in energy homeostasis. Front. Endocrinol..

[26] Liu J., Wang F., Ma Z., Wang X., Wang Y. (2011). Structural determination of three different series of compounds as Hsp90 inhibitors using 3D-QSAR modeling, molecular docking and molecular dynamics methods. Int. J. Mol. Sci..

[27] Yang H., Roth C.M., Ierapetritou M.G. (2009). A rational design approach for amino acid supplementation in hepatocyte culture. Biotechnol. Bioeng..

[28] Liu J., Zhang H., Xiao Z., Wang F., Wang X., Wang Y. (2011). Combined 3D-QSAR, molecular docking and molecular dynamics study on derivatives of peptide epoxyketone and tyropeptin-boronic acid as inhibitors against the β5 subunit of human 20S proteasome. Int. J. Mol. Sci..

[29] Sasmal S., Balaji G., Reddy H.R.K., Balasubrahmanyam D., Srinivas G., Kyasa S., Sasmal P.K., Khanna I., Talwar R., Suresh J. (2012). Design and optimization of quinazoline derivatives as melanin concentrating hormone receptor 1 (MCHR1) antagonists. Bioorg. Med. Chem. Lett..

[30] Sasmal S., Balasubrahmanyam D., Reddy H.R.K., Balaji G., Srinivas G., Cheera S., Abbineni C., Sasmal P.K., Khanna I., Sebastian V.J. (2012). Design and optimization of quinazoline derivatives as melanin concentrating hormone receptor 1 (MCHR1) antagonists. Bioorg. Med. Chem. Lett..

[31] Ulven T., Little P.B., Receveur J.-M., Frimurer F.M., Rist Ø., Nørregaard P.K., Högberg T. (2006). 6-Acylamino-2-amino-4-methylquinolines as potent melanin-concentrating hormone 1 receptor antagonists: Structure–activity exploration of eastern and western parts. Bioorg. Med. Chem. Lett..

[32] Ulven T., Frimurer T.M., Receveur J.-M., Little P.B., Rist Ø., Nørregaard P.K., Högberg T. (2005). 6-Acylamino-2-aminoquinolines as potent melanin-concentrating hormone 1 receptor antagonists. Identification, structure-activity relationship, and investigation of binding mode. J. Med. Chem..

[33] Ke Y.-Y., Shiao H.-Y., Hsu Y. C., Chu C.-Y., Wang W.-C., Lee Y.-C., Lin W.-H., Chen C.-H., Hsu J.T.A., Chang C.-W. (2013). 3D-QSAR-assisted drug design: Identification of a potent quinazoline-based aurora kinase inhibitor. Chem. Med. Chem..

[34] Bolden S., Boateng C.A., Zhu X.Y., Etukala J.R., Eyunni S.K., Jacobb M.R., Khan S.I., Ablordeppey S.Y. (2013). CoMFA studies and *in vitro* evaluation of some 3-substituted benzylthio quinolinium salts as anticryptococcal agents. Bioorg. Med. Chem..

[35] Böhm M., Stürzebecher J., Klebe G. (1999). Three-dimensional quantitative structure-activity relationship analyses using comparative molecular field analysis and comparative molecular similarity indices analysis to elucidate selectivity differences of inhibitors binding to trypsin, thrombin, and factor Xa. J. Med. Chem..

[36] Bringmann G., Rummey C. (2003). 3D QSAR investigations on antimalarial naphthylisoquinoline alkaloids by comparative molecular similarity indices analysis (CoMSIA), based on different alignment approaches. J. Chem. Inf. Comput. Sci..

[37] Da C., Mooberry S.L., Gupton J.T., Kellogg G.E. (2013). How to deal with low-resolution target structures: Using SAR, ensemble docking, hydropathic analysis, and 3D-QSAR to definitively map the αβ-tubulin colchicine site. J. Med. Chem..

[38] Tavares F.X., Al-Barazanji K.A., Bigham E.C., Bishop M.J., Britt C.S., Carlton D.L., Feldman P.L., Goetz A.S., Grizzle M.K., Guo Y.C. (2006). Potent, selective, and orally efficacious antagonists of melanin-concentrating hormone receptor 1. J. Med. Chem..

[39] Helal M.A., Chittiboyina A.G., Avery M.A. (2011). New insights into the binding mode of melanin concentrating hormone receptor-1 antagonists: Homology modeling and explicit membrane molecular dynamics simulation study. J. Chem. Inf. Model..

[40] Dixon R.A.F., Sigal I.S., Strader C.D. (1988). Structure-function analysis of the β-adrenergic receptor. Cold Spring Harb. Symp. Quant. Biol..

[41] Macdonald D., Murgolo N., Zhang R., Durkin J.P., Yao X., Strader C.D., Graziano M.P. (2000). Molecular characterization of the melanin concentrating hormone/receptor complex: Identification of critical residues involved in binding and activation. Mol. Pharmacol..

[42] Clark D.E., Higgs C., Wren S.P., Dyke H.J., Wong M., Norman D., Lockey P.M., Roach A.G. (2004). A virtual screening approach to finding novel and potent antagonists at the melanin-concentrating hormone 1 receptor. J. Med. Chem..

[43] Cirauqui N., Schrey A.K., Galiano S., Ceras J., Pérez-Silanes S., Aldana I., Monge A., Kühne R. (2010). Building a MCHR1 homology model provides insight into the receptor-antagonist contacts that are important for the development of new anti-obesity agents. Bioorg. Med. Chem..

[44] Shahlaeia M., Madadkar-Sobhani A., Mahnam K., Fassihi A., Saghaie L., Mansourian M. (2011). Homology modeling of human CCR5 and analysis of its binding properties through molecular docking and molecular dynamics simulation. Biochim. Biophys. Acta.

[45] Van Vlijmen H.W.T., Schaefer M., Karplus M. (1998). Improving the accuracy of protein pK_a_ calculations: Conformational averaging versus the average structure. Proteins.

[46] Witty D.R., Bateson J.H., Hervieu G.J., Jeffrey P., Johnson C.N., Muir A.I., O’Hanlon P.J., Stemp G., Stevens A.J., Thewlis K.M. (2006). SAR of biphenyl carboxamide ligands of the human melanin-concentrating hormone receptor 1 (MCHR1): Discovery of antagonist SB-568849. Bioorg. Med. Chem. Lett..

[47] Giordanetto F., Karlsson O., Lindberg J., Larsson L.-O., Linusson A., Evertsson E., Morgan D.G.A., Inghardt T. (2007). Discovery of cyclopentane- and cyclohexane-trans-1,3-diamines as potent melanin-concentrating hormone receptor 1 antagonists. Bioorg. Med. Chem. Lett..

[48] Cavasotto C.N., Orry A.J.W., Murgolo N.J., Czarniecki M.F., Kocsi S.A., Hawes B.E., O’Neill K.A., Hine H., Burton M.S., Voigt J.H. (2008). Discovery of novel chemotypes to a G-protein-coupled receptor through ligand-steered homology modeling and structure-based virtual screening. J. Med. Chem..

[49] Abu-Hammad A., Zalloum W.A., Zalloum H., Abu-Sheikha G., Taha M.O. (2009). Homology modeling of MCH1 receptor and validation by docking/scoring and protein-aligned CoMFA. Eur. J. Med. Chem..

[50] Sasmal P.K., Sasmal S., Rao P.T., Venkatesham B., Roshaiah M., Abbineni C., Khanna I., Jadhav V.P., Suresh J., Talwar R. (2010). Discovery of novel, orally available benzimidazoles as melanin concentrating hormone receptor 1 (MCHR1) antagonists. Bioorg. Med. Chem. Lett..

[51] Kamata M., Yamashita T., Imaeda T., Tanaka T., Terauchi J., Miyamoto M., Ora T., Tawada M., Endo S., Takekawa S. (2011). Discovery, synthesis, and structure–activity relationship of 6-aminomethyl-7,8-dihydronaphthalenes as human melanin-concentrating hormone receptor 1 antagonists. Bioorg. Med. Chem..

[52] Clark M., Cramer R.D., Vanopdenbosch N. (1989). Validation of the general purpose tripos 5.2 force field. J. Comput. Chem..

[53] Gasteiger J., Marsili M. (1980). Iterative partial equalization of orbital electronegativity-a rapid access to atomic charges. Tetrahedron.

[54] Wei S., Ji Z., Zhang H., Zhang J., Wang Y., Wu W. (2011). Isolation, biological evaluation and 3D-QSAR studies of insecticidal/narcotic sesquiterpene polyol esters. J. Mol. Model..

[55] Chang H.-W., Chung F.-S., Yang C.-N. (2013). Molecular modeling of p38αmitogen-activated protein kinase inhibitors through 3D-QSAR and molecular dynamics simulations. J. Chem. Inf. Model..

[56] Lan P., Chen W.-N., Chen W.-M. (2011). Molecular modeling studies on imidazo (4,5-*b*)pyridine derivatives as Aurora A kinase inhibitors using 3D-QSAR and docking approaches. Eur. J. Med. Chem..

[57] Pérez-Villanueva J., Medina-Franco J.L., Caulfield T.R., Hernández-Campos A., Hernández-Luis F., Yépez-Mulia L., Castillo R. (2011). Comparative molecularfield analysis (CoMFA) and comparative molecular similarity indices analysis (CoMSIA) of some benzimidazole derivatives with trichomonicidal activity. Eur. J. Med. Chem..

[58] Li X., Ye L., Wang X., Wang X., Liu H., Qian X., Zhu Y., Yu H. (2012). Molecular docking, molecular dynamics simulation, and structure-based 3D-QSAR studies on estrogenic activity of hydroxylated polychlorinated biphenyls. Sci. Total Environ..

[59] Mouchlis V.D., Melagraki G., Mavromoustakos T., Kollias G., Afantitis A. (2012). Molecular modelingon pyrimidine-urea inhibitors of TNF-α production: An integrated approach using a combination of molecular docking, classification techniques, and 3D-QSAR CoMSIA. J. Chem. Inf. Model..

[60] Jain E., Bairoch A., Duvaud S., Phan I., Redaschi N., Suzek B.E., Martin M.J., McGarvey P., Gasteiger E. (2009). Infrastructure for the life sciences: design and implementation of the UniProt website. BMC Bioinform..

[61] Cherezov V., Rosenbaum D.M., Hanson M.A., Rasmussen S.G., Thian F.S., Kobilka T.S., Choi H.J., Kuhn P., Weis W.I., Kobilka B.K. (2007). GPCR engineering yields high-resolution structural insights into β2-adrenergic receptor function. Science.

[62] Bordoli L., Kiefer F., Arnold K., Benkert P., Battey J., Schwede T. (2009). Protein structure homology modeling using SWISS-MODEL workspace. Nat. Protoc..

[63] Verdonk M.L., Cole J.C., Hartshorn M.J., Murray C.W., Taylor R.D. (2003). Improved protein–ligand docking using GOLD. Proteins.

[64] Tuccinardi T., Botta M., Giordano A., Martinelli A. (2010). Protein kinases: Docking and homology modeling reliability. J. Chem. Inf. Model..

[65] Arooj M., Sakkiah S., Kim S., Arulalapperumal V., Lee K.W. (2013). A combination of receptor-based pharmacophore modeling & QM techniques for identification of human chymase inhibitors. PLoS One.

[66] Karplus M., McCammon J.A. (2002). Molecular dynamics simulations of biomolecules. Nat. Struct. Biol..

[67] Spoel D.V.D., Lindahl E., Hess B., Groenhof G., Mark A.E., Berendsen H.J.C. (2005). GROMACS: Fast, flexible, and free. J. Comput. Chem..

[68] Schüttelkopf A.W., van Aalten D.M.F. (2004). PRODRG: A tool for high-throughput crystallography of protein-ligand complexes. Acta Crystallogr..

[69] Berendsen H.J.C., Postma J.P.M., Van Gunsteren W.F., Hermans J. (1981). Interaction models for water in relation to protein hydration. Intermolecular Forces.

[70] Lindahl E., Hess B., Spoel D.V.D. (2001). GROMACS 3.0: A package for molecular simulation and trajectory analysis. J. Mol. Model..

[71] Lin J.-H., Perryman A. L., Schames J. R., McCammon J. A. (2002). Computational drug design accommodating receptor flexibility: The relaxed complex scheme. J. Am. Chem. Soc..

[72] Ren S., Sato R., Hasegawa K., Ohta H., Masuda S. (2013). A predicted structure for the PixD−PixE complex determined by homology modeling, docking simulations, and a mutagenesis study. Biochemistry.

[73] Parrinello M., Rahman A. (1981). Polymorphic transitions in single crystals: A new molecular dynamics method. J. Appl. Phys..

[74] Skelton A.A., Maharaj Y.R., Soliman M.E.S. (2014). Target-bound generated pharmacophore model to improve the pharmacophore-based virtual screening: Identification of G-protein coupled human CCR2 receptors inhibitors as anti-Inflammatory drugs. Cell. Mol. Bioeng..

[75] Jo S., Kim T., Im W. (2007). Automated builder and database of protein/membrane complexes for molecular dynamics simulations. PLoS One.

