# Supplementary material for: Profiling the Interaction Mechanism of Quinoline/Quinazoline Derivatives as MCHR1 Antagonists: An in Silico Method
Source: Int J Mol Sci. 2014 Sep 1;15(9):15475–502. doi: 10.3390/ijms150915475 (PMC4200842; doi:10.3390/ijms150915475)
Supplement: Supplementary File 1 [file ijms-15-15475-s001.pdf]

Supplementary Information

Table S1. Structures and pIC_{50} values of compounds with skeleton type A in the data set.

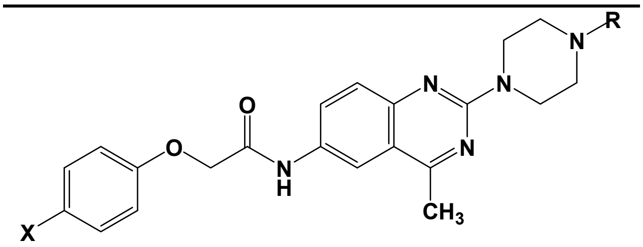

Skeleton type A

Compound	X	R	pIC_{50}(M)
**1**[#]	Cl	Me	6.66
**2**	Cl	Et	6.16
**3**	Cl	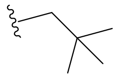	6.11
**4**	Cl	^{c}Pr	6.57
**5**	Cl	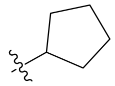	7.02
**6**	Cl	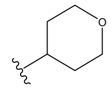	7.35
**7**	OCF_3	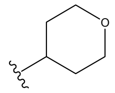	8.15
**8**	Cl	CO^{i}Pr	6.65
**9**	OCF_3	CO^{i}Pr	7.44
**10**	Cl	$COCH_2$^{i}Pr	6.85
**11**	Cl	$COCH_2OMe$	6.67
**12**	Cl	CO^{c}Pr	6.60
**13**[#]	OCF_3	CO^{c}Pr	7.31
**14**	Cl	CO^{c}Bu	6.49
**15**	OCF_3	CO^{c}Bu	7.08
**16**[#]	Cl	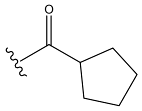	6.52
**17**[#]	Cl	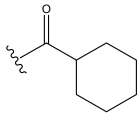	6.94
**18**	Cl	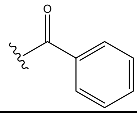	5.14

[#] Molecules belonging to the test set. ^{i} means iso-. ^{c} means cycloalkane.

Table S2. Structures and pIC_{50} values of compounds with skeleton type B in the data set.

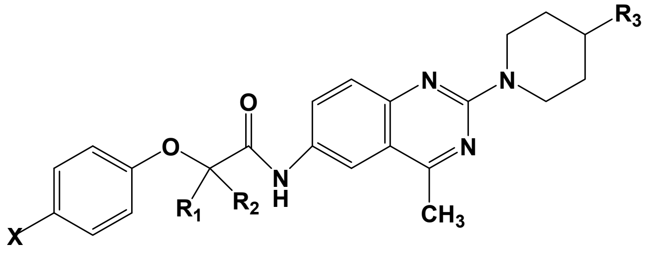

Skeleton type B

Compound	X	R_1	R_2	R_3	pIC_{50}(M)
**19**[#]	Cl	H	H	H	6.74
**20**[#]	Cl	H	H	OH	6.82
**21**	Cl	H	H	OMe	6.67
**22**	Cl	H	H	CONH^{i}Pr	7.82
**23**[#]	OCF_3	H	H	CONH^{i}Pr	7.77
**24**	OCF_3	H	H	$CONMe_2$	7.33
**25**	OCF_3	H	H	CONH^{c}Pr	8.05
**26**[#]	OCF_3	H	H	$CONH_2$	7.64
**27**[#]	OCF_3	H	H	CO^{c}Pr	8.00
**28**	Cl	H	H	NH_2	6.82
**29**	OCF_3	H	H	NH_2	7.66
**30**[#]	Cl	H	H	NHCO^{i}Pr	7.82
**31**	Cl	H	H	NHCONH^{i}Pr	8.15
**32**[#]	Cl	H	H	Pyrrolidine	7.70
**33**[#]	Cl	Me	H	Pyrrolidine	6.59
**34**	OCF_3	H	H	Pyrrolidine	7.51
**35**	OCF_3	H	H	Morpholine	8.22
**36**	OCF_3	H	H	2−Pyrrolidinone	7.72

[#] Molecules belonging to the test set.

Table S3. Structures and pIC_{50} values of compounds with skeleton type C in the data set.

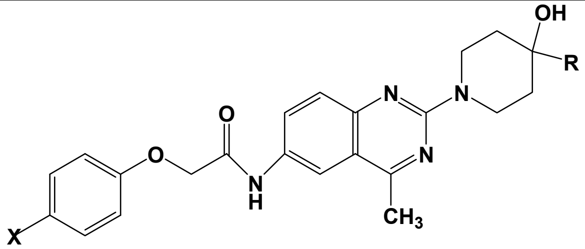

Skeleton type C

Compound	X	R	pIC_{50}(M)
**37**	Cl	Me	6.63
**38**	Cl	Et	6.68
**39**	OCF_3	Et	6.94
**40**	OCF_3	*c*−Pr	7.00
**41**	OCF_3	*c*−Bu	7.10

Table S3. *Cont.*

Compound	X	R	pIC_{50}(M)
**42**	OCF_3	*c*−Hexyl	6.49
**43**	OCF_3	$CONH_2$	6.20
**44**[#]	OCF_3	Ph	6.45
**45**	OCF_3	*p*−F−phenyl	6.82
**46**[#]	OCF_3	*m*−F−phenyl	6.20
**47**	OCF_3	5−F−2−pyridyl	6.67

[#] Molecules belonging to the test set.

Table S4. Structures and pIC_{50} values of compounds with skeleton type D in the data set.

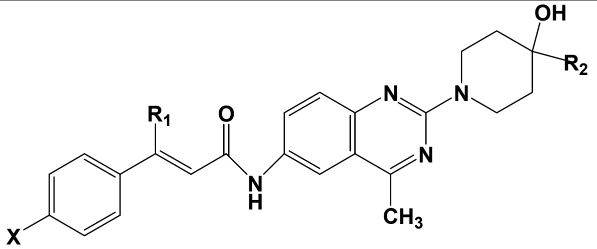

Skeleton type D

Compound	X	R_1	R_2	pIC_{50}(M)
**48**	OCF_3	H	H	7.21
**49**[#]	Cl	H	H	6.97
**50**[#]	Cl	Me	H	6.17
**51**[#]	OCF_3	H	^{c}Pr	6.68
**52**	OCF_3	Me	^{c}Pr	6.45
**53**	Cl	H	^{c}Pr	6.83
**54**	Cl	Me	^{c}Pr	6.27
**55**[#]	Me	H	^{c}Pr	6.46
**56**[#]	CF_3	H	^{c}Pr	6.63
**57**[#]	OMe	H	^{c}Pr	6.94
**58**	CHF_2	H	^{c}Pr	6.91
**59**[#]	$OCHF_2$	H	^{c}Pr	6.70
**60**	o,p−DiCl	H	^{c}Pr	6.21
**61**	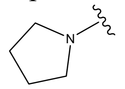	H	^{c}Pr	5.74
**62**	Cl	H	Ph	5.96
**63**	Cl	H	^{c}Bu	6.69
**64**[#]	Cl	H	^{i}Pr	6.77
**65**[#]	Cl	H	^{n}Pr	6.61
**66**[#]	Cl	H	Et	6.57
**67**[#]	Cl	H	CH_2Ac	6.82
**68**	Cl	H	$(CH_2)_2OH$	6.66
**69**[#]	Cl	H	(CH2)2OMe	6.61

[#] Molecules belonging to the test set. [n] means neo-.

Table S5. Structures and pIC$_{50}$ values of compounds with skeleton type E in the data set.

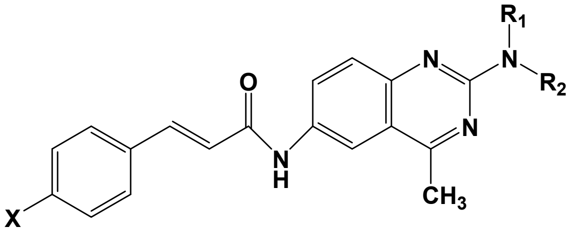

Skeleton type E

Compound	X	R$_1$	R$_2$	pIC$_{50}$(M)
**70**[#]	Cl	*c*–Pentyl	Me	6.40
**71**	Cl	*c*–Pentyl	$(CH_2)_2OH$	6.07
**72**[#]	Cl	H	$(CH_2)_2OH$	6.26
**73**	OCF_3	H	$(CH_2)_3OH$	7.44
**74**[#]	OCF_3	^{i}Pr	$(CH_2)_3OH$	6.54
**75**[#]	OCF_3	H	$(CH_2)_2$NHMs	6.77
**76**	OCF_3	H	$(CH_2)_3$NHMs	7.27
**77**	OCF_3	H	$(CH_2)_2$NHCO^{c}Pr	7.21
**78**	OCF_3	H	$(CH_2)_3$NHCO^{c}Pr	7.20
**79**	OCF_3	H	$(CH_2)_3$CONH^{c}Pr	7.70

[#] Molecules belonging to the test set.

Table S6. Structures and pIC$_{50}$ values of compounds with skeleton type F in the data set.

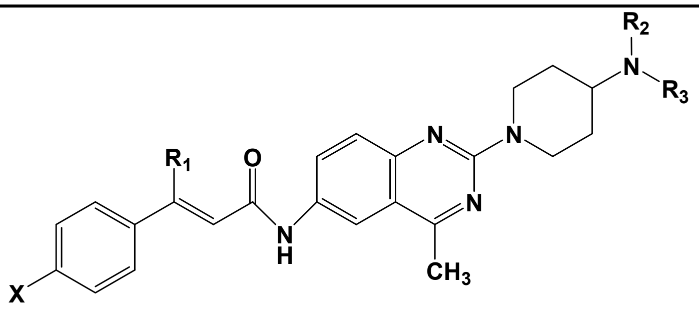

Skeleton type F

Compound	X	R$_1$	R$_2$	R$_3$	pIC$_{50}$(M)
**80**	Cl	H	$-(CH_2)_2O(CH_2)_2-$		7.80
**81**	OCF_3	H	$-(CH_2)_2O(CH_2)_2-$		8.00
**82**	OCF_3	H	$-COCH_2O(CH_2)_2-$		7.52
**83**	OCF_3	H	$-CO(CH_2)_4-$		7.29
**84**	OCF_3	H	$-CO(CH_2)_3-$		7.96
**85**	OCF_3	Me	$-CO(CH_2)_3-$		7.16
**86**	Cl	H	$-CO(CH_2)_3-$		7.13
**87**	Cl	Me	$-CO(CH_2)_3-$		7.09
**88**	OCF_3	H	$-SO_2(CH_2)_3-$		8.40
**89**[#]	OCF_3	H	$-CO(CH_2)_2CO-$		7.03
**90**	OCF_3	H	$-COO(CH_2)_2-$		7.39

Table S6. *Cont.*

Compound	X	R_1	R_2	R_3	pIC_{50}(M)
**91**#	OCF_3	H	$-COOCMe_2CH_2-$		7.17
**92**	OCF_3	H	$-CONH(CH_2)_2-$		7.18
**93**	OCF_3	H	$-CONMe(CH_2)_2-$		7.68
**94**#	OCF_3	H	$-SO_2NH(CH_2)_2-$		6.84
**95**	OCF_3	H	$-SO_2NMe(CH_2)_2-$		6.55
**96**	Cl	H	H	$CO^{c}Pr$	6.65
**97**	Cl	H	H	SO_2Me	7.51
**98**	Cl	Me	H	SO_2Me	6.95
**99**	Cl	H	H	$COCH_2OH$	7.39
**100**#	Cl	H	H	$COCH_2OMe$	7.35
**101**	OCF_3	H	H	$COCH_2OH$	7.59
**102**#	OCF_3	H	H	$COCH_2OMe$	7.49
**103**	OCF_3	H	Me	$COCH_2OH$	7.35
**104**	OCF_3	H	Me	$COCH_2OH$	7.04
**105**	OCF_3	H	H	SO_2NMe_2	6.98

Molecules belonging to the test set.

Table S7. Structures and pIC_{50} values of compounds with skeleton type H in the data set.

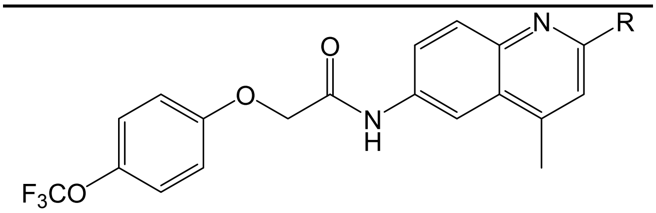

Skeleton type G

Compound	R	pIC_{50}(M)
**106**	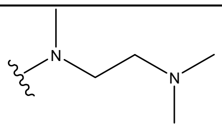	7.74
**107**	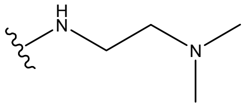	7.74
**108**	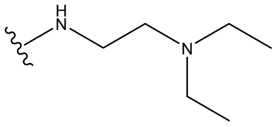	7.82
**109**	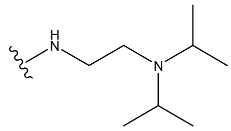	7.66
**110**	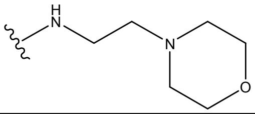	8.02

Table S7. *Cont.*

Compound	R	pIC50(M)
**111**#	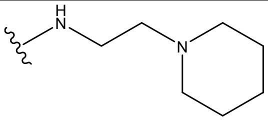	8.28
**112**	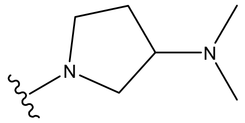	8.21
**113**#	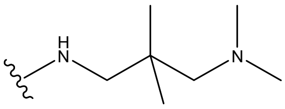	7.80
**114**	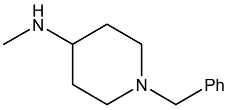	7.40
**115**	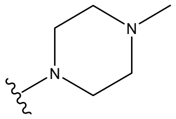	7.54
**116**#	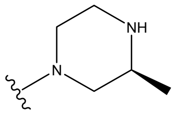	7.89
**117**	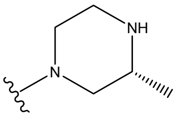	7.74
**118**	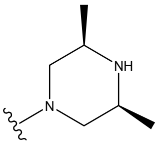	7.06
**119**	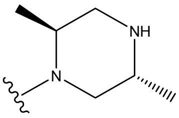	7.70
**120**	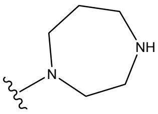	7.92
**121**	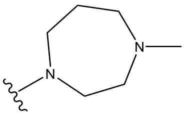	7.70
**122**	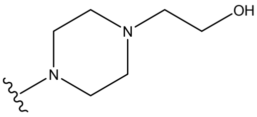	8.30

Molecules belonging to the test set.

Table S8. Structures and pIC_{50} values of compounds with skeleton type I in the data set.

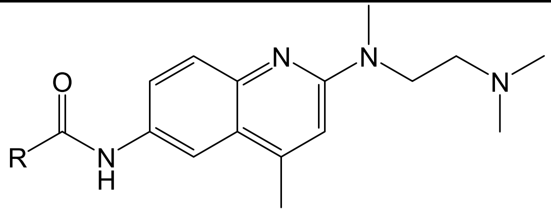

Skeleton type H

Compound	R	pIC_{50}(M)
**123**	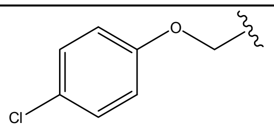	7.08
**124**	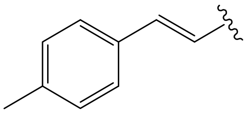	7.32
**125**[#]	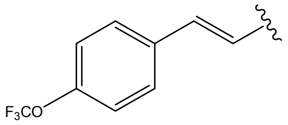	7.72
**126**[#]	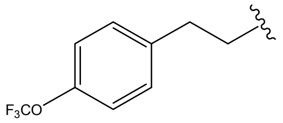	7.40
**127**[#]	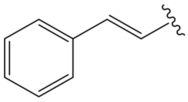	5.16
**128**	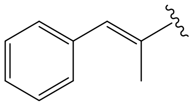	5.02
**129**	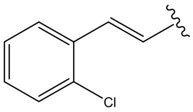	5.60
**130**	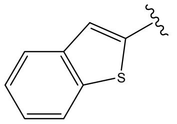	5.68
**131**	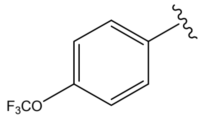	5.56
**132**[#]	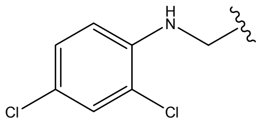	6.90
**133**	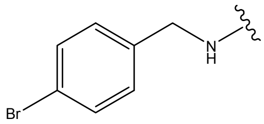	6.57
**134**	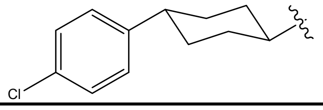	6.37

Table S8. *Cont.*

Compound	R	pIC50(M)
**135**	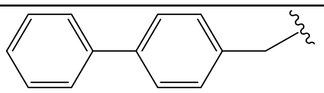	5.48
**136**	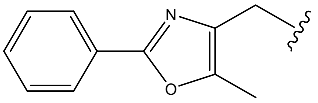	6.25
**137**	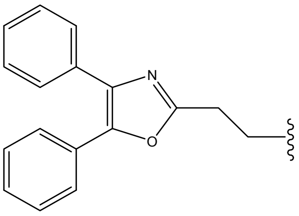	6.79
**138**	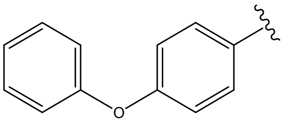	6.68
**139**	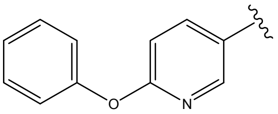	6.98
**140**	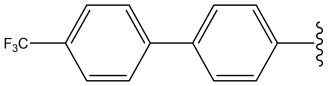	7.20
**141**	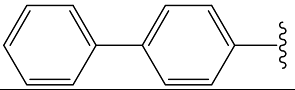	7.38

[#] Molecules belonging to the test set.

Table S9. Structures and pIC_{50} values of compounds with skeleton type J in the data set.

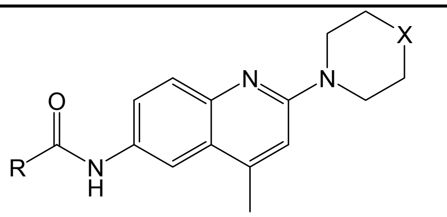

Skeleton type I

Compound	R	X	pIC50(M)
**142**	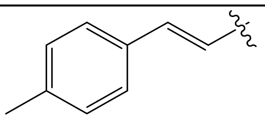	NMe	7.35
**143**[#]	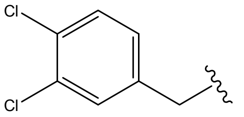	NEt	6.14
**144**	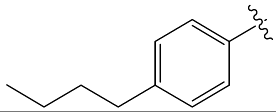	NEt	6.94

Table S9. *Cont.*

Compound	R	X	pIC50(M)
**145**[#]	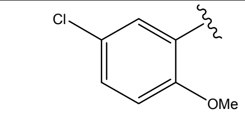	NEt	5.58
**146**	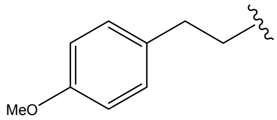	NEt	6.60
**147**[#]	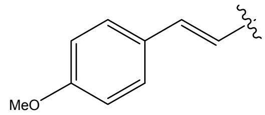	NEt	7.80
**148**[#]	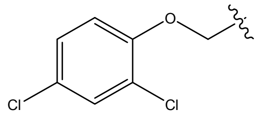	Net	7.62
**149**[#]	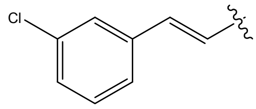	NMe	5.74
**150**[#]	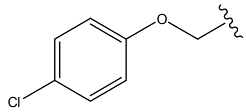	Net	7.66
**151**	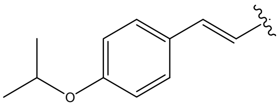	NEt	7.02
**152**[#]	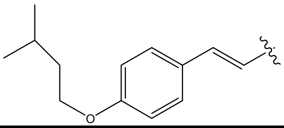	NMe	6.36

[#] Molecules belonging to the test set.

Table S10. Structures and pIC_{50} values of compounds with skeleton type K in the data set.

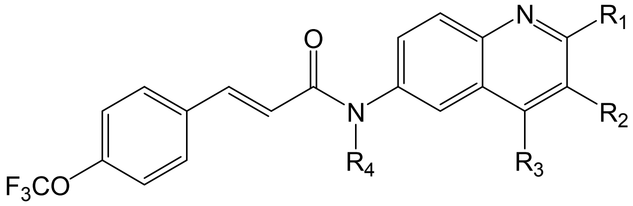

Skeleton type J

Compound	R1	R2	R3	R4	pIC_{50}(M)
**153**	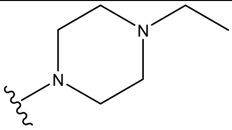	H	Me	H	7.80
**154**	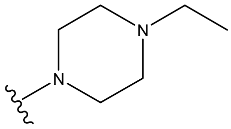	H	Me	H	6.46

Table S10. *Cont.*

Compound	R1	R2	R3	R4	pIC50(M)
**155**	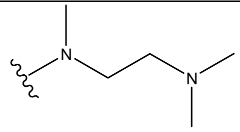	H	H	H	6.76
**156**[#]	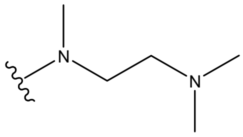	H	H	H	6.09
**157**	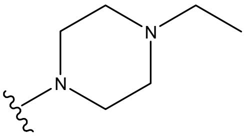	Me	H	H	6.32
**158**[#]	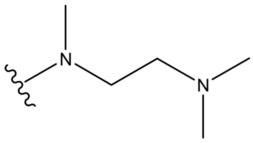	Me	H	H	5.98
**159**	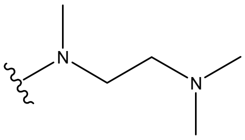	H	Et	H	6.62
**160**[#]	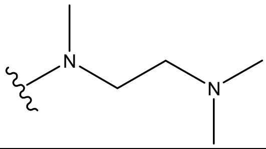	H	Me	Me	5.96

[#] Molecules belonging to the test set.

Table S11. Structures and pIC_{50} values of compounds with skeleton type L in the data set.

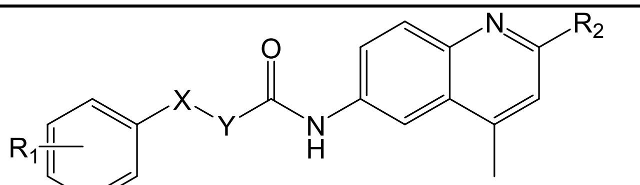

Skeleton type K

Compound	R_1	X–Y	R_2	pIC_{50}(M)
**161**	2,4–Cl_2	O–CH_2	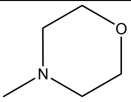	7.23
**162**	4–CF_3O	O–CH_2	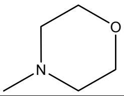	7.09

Table S11. *Cont.*

Compound	R1	X−Y	R2	pIC50(M)
**163**#	2,4−Cl_2	O−CH_2	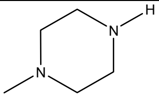	7.80
**164**	4−CF_3O	CH=CH	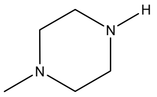	7.19
**165**#	4−CF_3O	CH=CH	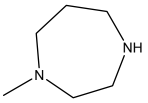	7.64
**166**	4−CF_3O	CH=CH	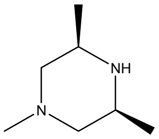	6.39
**167**	4−CF_3O	O−CH_2	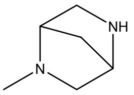	8.01
**168**#	4−CF_3O	O−CH_2	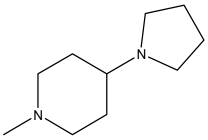	8.15
**169**	4−CF_3O	O−CH_2	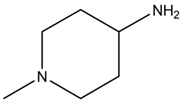	8.46
**170**#	4−CF_3O	O−CH_2	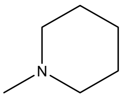	7.01
**171**#	2,4−Cl_2	O−CH_2	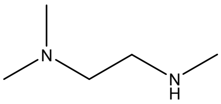	8.16
**172**	4−CF_3O	CH=CH	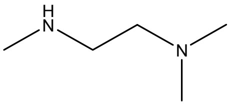	7.89
**173**	4−CF_3O	CH=CH	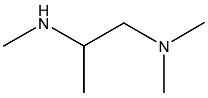	7.66
**174**	4−CF_3O	O−CH_2	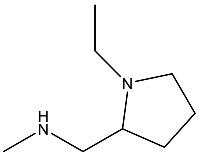	7.68
**175**	4−CF_3O	O−CH_2	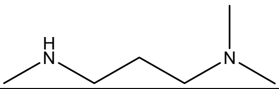	8.42

Table S11. *Cont.*

Compound	R_1	X−Y	R_2	pIC_{50}(M)
**176**#	2,4−Cl_2	O−CH_2	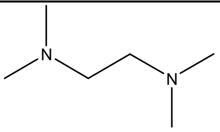	8.03
**177**#	2,4−Cl_2	O−CH_2	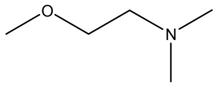	6.74
**178**	4−CF_3O	CH=CH	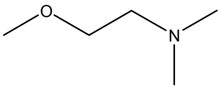	6.38
**179**	2,4−Cl_2	O−CH_2	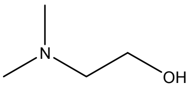	7.37
**180**#	4−CF_3O	CH=CH	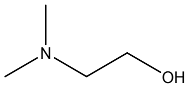	7.39
**181**	2,4−Cl_2	O−CH_2	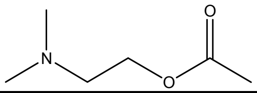	7.54

Molecules belonging to the test set.

Table S12. Q^2, R^2_{ncv} and R^2_{pred} of all 34 possible combinations of different descriptor fields.

No.	PLS Statistics	Q^2	R^2_{ncv}	R^2_{pred}
	CoMFA			
1	*S*	0.280	0.784	0.265
2	*E*	0.296	0.644	0.262
3	*SE*	0.372	0.896	0.544
	CoMSIA			
4	S	0.206	0.405	−0.211
5	*E*	0.321	0.628	0.225
6	*H*	0.368	0.662	0.514
7	*D*	0.234	0.499	0.074
8	*A*	0.169	0.368	−0.363
9	*SE*	0.319	0.658	−0.044
10	*SH*	0.376	0.677	0.459
11	*SD*	0.093	0.498	0.381
12	*SA*	0.254	0.772	0.501
13	*EH*	0.368	0.736	0.498
14	*ED*	0.302	0.687	0.425
15	*EA*	0.412	0.713	0.460
16	*HD*	0.397	0.757	0.639
17	*HA*	0.463	0.874	0.643

Table S12. *Cont.*

No.	PLS Statistics	Q^2	R^2_{ncv}	R^2_{pred}
18	*DA*	0.204	0.648	0.264
19	*SEH*	0.389	0.689	0.536
20	*SED*	0.288	0.655	0.448
21	*SEA*	0.386	0.831	0.488
22	*SHD*	0.398	0.771	0.662
23	*SHA*	0.453	0.868	0.665
24	*SDA*	0.413	0.837	0.609
25	*EHD*	0.405	0.801	0.616
26	*EHA*	0.434	0.886	0.635
27	*EDA*	0.446	0.834	0.514
28	*HDA*	0.477	0.889	0.576
29	*SEHD*	0.404	0.805	0.658
30	*SEHA*	0.428	0.875	0.666
31	*SEDA*	0.454	0.861	0.582
32	*SHDA*	0.509	0.841	0.745
33	*EHDA*	0.476	0.838	0.687
34	*SEHDA*	0.469	0.890	0.657

Table S13. Observed and predicted MCHR1 antagonist activities (pIC_{50}, M).

Compound	Observed Activities	CoMFA		CoMSIA	
		Predicted	Residue	Predicted	Residue
**1**	6.66	6.511	−0.149	6.583	−0.077
**2**	6.16	6.477	0.317	6.496	0.336
**3**	6.11	6.169	0.059	6.054	−0.056
**4**	6.57	6.464	−0.106	6.471	−0.099
**5**	7.02	7.096	0.076	6.66	−0.36
**6**	7.35	7.309	−0.041	7.15	−0.2
**7**	8.15	8.075	−0.075	7.959	−0.191
**8**	6.65	7.01	0.36	6.652	0.002
**9**	7.44	7.227	−0.213	6.855	−0.585
**10**	6.85	6.729	−0.121	6.491	−0.359
**11**	6.67	6.789	0.119	6.556	−0.114
**12**	6.6	6.648	0.048	6.615	0.015
**13**	7.31	7.016	−0.294	6.821	−0.489
**14**	6.49	6.557	0.067	6.673	0.183
**15**	7.08	7.336	0.256	7.398	0.318
**16**	6.52	6.255	−0.265	6.596	0.076
**17**	6.94	7.027	0.087	6.623	−0.317
**18**	5.14	4.603	−0.537	5.063	−0.077
**19**	6.74	6.956	0.216	7.016	0.276
**20**	6.82	7.12	0.3	6.928	0.108
**21**	6.67	6.902	0.232	6.951	0.281
**22**	7.82	7.926	0.106	7.684	−0.136
**23**	7.77	8.059	0.289	7.803	0.033

Table S13. *Cont.*

Compound	Observed Activities	CoMFA		CoMSIA	
		Predicted	Residue	Predicted	Residue
**24**	7.33	7.227	−0.103	7.182	−0.148
**25**	8.05	8.116	0.066	7.897	−0.153
**26**	7.64	7.529	−0.111	7.966	0.326
**27**	8	7.633	−0.367	7.153	−0.847
**28**	6.82	6.932	0.112	7.31	0.49
**29**	7.66	7.152	−0.508	7.499	−0.161
**30**	7.82	7.547	−0.273	7.365	−0.455
**31**	8.15	8.278	0.128	8.002	−0.148
**32**	7.7	7.024	−0.676	7.196	−0.504
**33**	6.59	6.079	−0.511	6.565	−0.025
**34**	7.51	7.054	−0.456	7.323	−0.187
**35**	8.22	8.123	−0.097	7.948	−0.272
**36**	7.72	7.799	0.079	7.848	0.128
**37**	6.63	6.784	0.154	6.916	0.286
**38**	6.68	6.615	−0.065	6.823	0.143
**39**	6.94	6.755	−0.185	7.024	0.084
**40**	7	6.9	−0.1	7.003	0.003
**41**	7.1	6.96	−0.14	6.813	−0.287
**42**	6.49	6.873	0.383	6.631	0.141
**43**	6.2	6.36	0.16	6.031	−0.169
**44**	6.45	6.994	0.544	6.898	0.448
**45**	6.82	6.916	0.096	7.116	0.296
**46**	6.2	6.523	0.323	6.086	−0.114
**47**	6.67	6.651	−0.019	6.82	0.15
**48**	7.21	7.131	−0.079	7.099	−0.111
**49**	6.97	6.643	−0.327	6.802	−0.168
**50**	6.17	6.331	0.161	6.585	0.415
**51**	6.68	7.152	0.472	6.957	0.277
**52**	6.45	6.629	0.179	6.875	0.425
**53**	6.83	6.584	−0.246	6.627	−0.203
**54**	6.27	6.057	−0.213	6.452	0.182
**55**	6.46	7.027	0.567	6.633	0.173
**56**	6.63	6.545	−0.085	6.796	0.166
**57**	6.94	6.872	−0.068	6.62	−0.32
**58**	6.91	6.805	−0.105	6.448	−0.462
**59**	6.7	6.218	−0.482	6.58	−0.12
**60**	6.21	6.153	−0.057	5.904	−0.306
**61**	5.74	5.902	0.162	5.668	−0.072
**62**	5.96	6.058	0.098	6.265	0.305
**63**	6.69	6.499	−0.191	6.635	−0.055
**64**	6.77	6.336	−0.434	6.449	−0.321
**65**	6.61	6.749	0.139	6.481	−0.129
**66**	6.57	6.34	−0.23	6.602	0.032

Table S13. *Cont.*

Compound	Observed Activities	CoMFA		CoMSIA	
		Predicted	Residue	Predicted	Residue
**67**	6.82	6.031	−0.789	6.825	0.005
**68**	6.66	6.476	−0.184	6.716	0.056
**69**	6.61	6.59	−0.02	6.937	0.327
**70**	6.4	6.213	−0.187	6.507	0.107
**71**	6.07	6.257	0.187	6.154	0.084
**72**	6.26	6.8	0.54	6.742	0.482
**73**	7.44	7.421	−0.019	7.19	−0.25
**74**	6.54	6.677	0.137	6.739	0.199
**75**	6.77	7.204	0.434	6.978	0.208
**76**	7.27	7.185	−0.085	7.119	−0.151
**77**	7.21	7.279	0.069	7.047	−0.163
**78**	7.2	7.213	0.013	6.976	−0.224
**79**	7.7	7.536	−0.164	7.672	−0.028
**80**	7.8	7.708	−0.092	7.796	−0.004
**81**	8	8.14	0.14	8.058	0.058
**82**	7.52	7.181	−0.339	7.298	−0.222
**83**	7.29	7.49	0.2	7.333	0.043
**84**	7.96	7.896	−0.064	7.451	−0.509
**85**	7.16	7.431	0.271	7.336	0.176
**86**	7.13	7.055	−0.075	7.231	0.101
**87**	7.09	6.903	−0.187	6.964	−0.126
**88**	8.4	7.995	−0.405	7.978	−0.422
**89**	7.03	6.942	−0.088	7.19	0.16
**90**	7.39	7.307	−0.083	7.643	0.253
**91**	7.17	7.536	0.366	7.69	0.52
**92**	7.18	7.696	0.516	6.973	−0.207
**93**	7.68	7.844	0.164	7.74	0.06
**94**	6.84	7.933	1.093	7.025	0.185
**95**	6.55	6.92	0.37	7	0.45
**96**	6.65	6.659	0.009	6.785	0.135
**97**	7.51	7.342	−0.168	7.501	−0.009
**98**	6.95	6.9	−0.05	7.295	0.345
**99**	7.39	7.465	0.075	7.442	0.052
**100**	7.35	6.61	−0.74	7.102	−0.248
**101**	7.59	7.736	0.146	7.584	−0.006
**102**	7.49	7.491	0.001	7.427	−0.063
**103**	7.35	7.052	−0.298	7.394	0.044
**104**	7.04	7.014	−0.026	7.257	0.217
**105**	6.98	7.047	0.067	7.208	0.228
**106**	7.74	7.618	−0.122	7.381	−0.359
**107**	7.74	7.955	0.215	8.103	0.363
**108**	7.82	7.497	−0.323	7.417	−0.403
**109**	7.66	7.623	−0.037	7.9	0.24

Table S13. *Cont.*

Compound	Observed Activities	CoMFA		CoMSIA	
		Predicted	Residue	Predicted	Residue
**110**	8.02	8.283	0.263	8.494	0.474
**111**	8.28	8.346	0.066	8.292	0.012
**112**	8.21	7.872	−0.338	7.679	−0.531
**113**	7.8	7.635	−0.165	8.138	0.338
**114**	7.4	7.444	0.044	7.391	−0.009
**115**	7.54	7.697	0.157	7.69	0.15
**116**	7.89	7.709	−0.181	7.686	−0.204
**117**	7.74	7.768	0.028	7.648	−0.092
**118**	7.06	7.097	0.037	7.003	−0.057
**119**	7.7	7.681	−0.019	7.63	−0.07
**120**	7.92	7.843	−0.077	8.091	0.171
**121**	7.7	7.564	−0.136	7.546	−0.154
**122**	8.3	8.307	0.007	8.425	0.125
**123**	7.08	6.793	−0.287	7.071	−0.009
**124**	7.32	6.997	−0.323	6.723	−0.597
**125**	7.72	7.807	0.087	7.173	−0.547
**126**	7.4	7.017	−0.383	6.874	−0.526
**127**	5.16	6.374	1.214	5.862	0.702
**128**	5.02	5.427	0.407	5.949	0.929
**129**	5.6	5.737	0.137	5.653	0.053
**130**	5.68	5.937	0.257	6.262	0.582
**131**	5.56	5.749	0.189	5.606	0.046
**132**	6.9	5.842	−1.058	6.509	−0.391
**133**	6.57	6.318	−0.252	6.868	0.298
**134**	6.37	6.136	−0.234	6.367	−0.003
**135**	5.48	5.431	−0.049	5.4	−0.08
**136**	6.25	6.387	0.137	6.275	0.025
**137**	6.79	6.896	0.106	7.37	0.58
**138**	6.68	6.74	0.06	6.543	−0.137
**139**	6.98	6.916	−0.064	6.788	−0.192
**140**	7.2	7.299	0.099	7.193	−0.007
**141**	7.38	7.093	−0.287	6.823	−0.557
**142**	7.35	7.009	−0.341	6.825	−0.525
**143**	6.14	6.546	0.406	6.373	0.233
**144**	6.94	7.104	0.164	7.003	0.063
**145**	5.58	5.924	0.344	5.836	0.256
**146**	6.6	6.721	0.121	6.761	0.161
**147**	7.8	8.482	0.682	7.255	−0.545
**148**	7.62	7.438	−0.182	7.686	0.066
**149**	5.74	6.483	0.743	6.4	0.66
**150**	7.66	7.257	−0.403	7.54	−0.12
**151**	7.02	7.121	0.101	6.979	−0.041
**152**	6.36	7.246	0.886	6.971	0.611

Table S13. *Cont.*

Compound	Observed Activities	CoMFA		CoMSIA	
		Predicted	Residue	Predicted	Residue
**153**	7.8	7.066	−0.734	7.049	−0.751
**154**	6.46	6.895	0.435	6.951	0.491
**155**	6.76	6.574	−0.186	6.557	−0.203
**156**	6.09	6.799	0.709	6.323	0.233
**157**	6.32	6.628	0.308	6.866	0.546
**158**	5.98	6.363	0.383	6.254	0.274
**159**	6.62	6.877	0.257	6.709	0.089
**160**	5.96	6.875	0.915	6.459	0.499
**161**	7.23	7.207	−0.023	7.17	−0.06
**162**	7.09	7.497	0.407	7.527	0.437
**163**	7.8	7.389	−0.411	7.35	−0.45
**164**	7.19	7.137	−0.053	7.05	−0.14
**165**	7.64	7.185	−0.455	7.232	−0.408
**166**	6.39	6.866	0.476	6.733	0.343
**167**	8.01	7.911	−0.099	7.946	−0.064
**168**	8.15	7.997	−0.153	7.765	−0.385
**169**	8.46	8.032	−0.428	8.511	0.051
**170**	7.01	6.898	−0.112	7.277	0.267
**171**	8.16	7.339	−0.821	7.802	−0.358
**172**	7.89	7.515	−0.375	7.481	−0.409
**173**	7.66	7.526	−0.134	7.442	−0.218
**174**	7.68	7.534	−0.146	7.891	0.211
**175**	8.42	8.51	0.09	8.201	−0.219
**176**	8.03	7.875	−0.155	7.5	−0.53
**177**	6.74	6.365	−0.375	6.335	−0.405
**178**	6.38	6.77	0.39	6.564	0.184
**179**	7.37	7.575	0.205	7.635	0.265
**180**	7.39	7.224	−0.166	6.908	−0.482
**181**	7.54	7.864	0.324	7.657	0.117
